# Artificial Intelligence in the Diagnostic Use of Transcranial Doppler and Sonography: A Scoping Review of Current Applications and Future Directions

**DOI:** 10.3390/bioengineering12070681

**Published:** 2025-06-21

**Authors:** Giuseppe Miceli, Maria Grazia Basso, Elena Cocciola, Antonino Tuttolomondo

**Affiliations:** 1 Department of Health Promotion, Mother and Child Care, Internal Medicine and Medical Specialties (ProMISE) Università degli Studi di Palermo, Piazza delle Cliniche 2, 90127 Palermo, Italy; mariagbasso.92@gmail.com (M.G.B.);; 2 Internal Medicine and Stroke Care Ward, University Hospital, Policlinico “P. Giaccone”, 90100 Palermo, Italy

**Keywords:** artificial intelligence, Transcranial Doppler, sonography, neurovascular imaging, machine learning, deep learning

## Abstract

Artificial intelligence (AI) is revolutionizing the field of medical imaging, offering unprecedented capabilities in data analysis, image interpretation, and decision support. Transcranial Doppler (TCD) and Transcranial Color-Coded Doppler (TCCD) are widely used, non-invasive modalities for evaluating cerebral hemodynamics in acute and chronic conditions. Yet, their reliance on operator expertise and subjective interpretation limits their full potential. AI, particularly machine learning and deep learning algorithms, has emerged as a transformative tool to address these challenges by automating image acquisition, optimizing signal quality, and enhancing diagnostic accuracy. Key applications reviewed include the automated identification of cerebrovascular abnormalities such as vasospasm and embolus detection in TCD, AI-guided workflow optimization, and real-time feedback in general ultrasound imaging. Despite promising advances, significant challenges remain, including data standardization, algorithm interpretability, and the integration of these tools into clinical practice. Developing robust, generalizable AI models and integrating multimodal imaging data promise to enhance diagnostic and prognostic capabilities in TCD and ultrasound. By bridging the gap between technological innovation and clinical utility, AI has the potential to reshape the landscape of neurovascular and diagnostic imaging, driving advancements in personalized medicine and improving patient outcomes. This review highlights the critical role of interdisciplinary collaboration in achieving these goals, exploring the current applications and future directions of AI in TCD and TCCD imaging. This review included 41 studies on the application of artificial intelligence (AI) in neurosonology in the diagnosis and monitoring of vascular and parenchymal brain pathologies. Machine learning, deep learning, and convolutional neural network algorithms have been effectively utilized in the analysis of TCD and TCCD data for several conditions. Conversely, the application of artificial intelligence techniques in transcranial sonography for the assessment of parenchymal brain disorders, such as dementia and space-occupying lesions, remains largely unexplored. Nonetheless, this area holds significant potential for future research and clinical innovation.

## 1. Introduction

Artificial intelligence (AI) has emerged as a transformative force across numerous healthcare domains, particularly medical imaging [1]. Transcranial Doppler (TCD) and ultrasound represent critical diagnostic tools in neurosonology due to their non-invasive nature, real-time imaging capabilities, and cost-effectiveness [2]. TCD plays a pivotal role in the evaluation of cerebral hemodynamics and the detection of vascular pathologies. Transcranial Color-Coded Doppler ultrasound (TCCD), instead, extends the capabilities of traditional TCD by integrating B-mode imaging with Doppler flow assessment, enabling the enhanced visualization of the main intracranial vessels, such as the middle cerebral artery, anterior cerebral artery, posterior cerebral artery, and vertebra–basal circulation, improving the spatial resolution of cerebrovascular evaluations [3]. However, both modalities are inherently limited by their reliance on operator expertise, subjective interpretation, and potential variability in image quality [4]. Recent advancements in AI, particularly in machine learning (ML) and deep learning (DL), offer novel opportunities to address these limitations by automating image analysis, standardizing diagnostic workflows, and improving overall accuracy. This scoping review provides a comprehensive overview of the current diagnostic applications of AI in TCD and ultrasound, emphasizing its potential to enhance clinical decision-making and streamline diagnostic processes. Furthermore, this review explores the challenges associated with the integration of AI into routine clinical practice, such as data standardization, algorithm transparency, and regulatory considerations. By analyzing existing evidence and identifying future directions, this article aims to highlight the transformative potential of AI in augmenting the diagnostic utility of TCD and ultrasound.

## 2. Research Strategy

**Figure 1:**
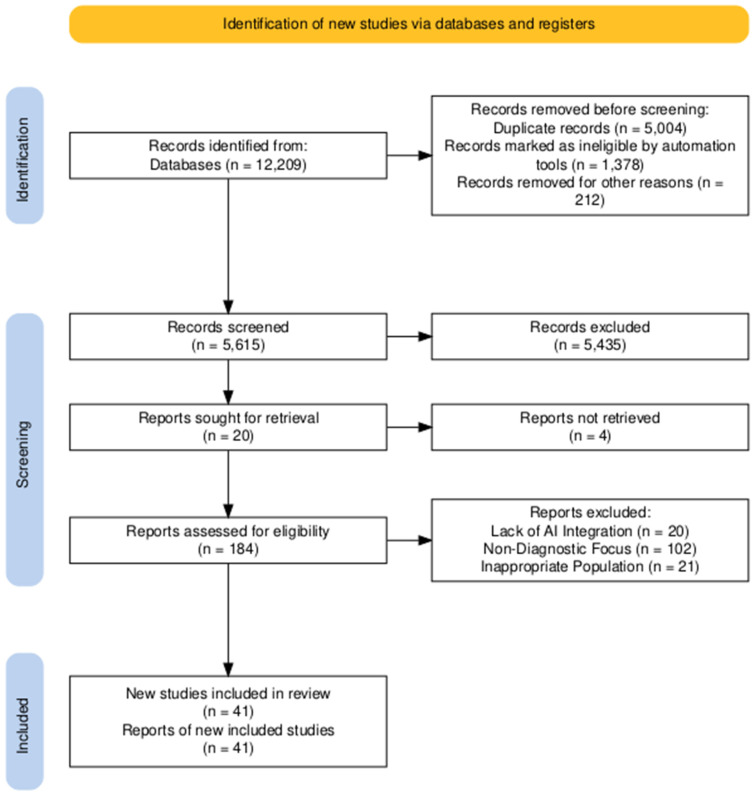
Research flow diagram according to PRISMA methodology.

A comprehensive literature search was conducted to identify relevant studies on the applications of AI in TCD and ultrasound. The search was performed using the following academic databases: *PubMed*, *IEEE Xplore*, *Scopus*, *Web of Science*, and *Google Scholar*. A combination of keywords and Medical Subject Heading (MeSH) terms was employed to ensure a broad yet focused retrieval of relevant articles. The search strategy included the following Boolean combinations: (“Artificial Intelligence” OR “Machine Learning” OR “Deep Learning” OR “Neural Networks” OR “ Support Vector Machines” OR “Recurrent Neural Networks” OR “Automated emboli detection”) AND (“Transcranial Doppler” OR “TCD” OR “Cerebrovascular Ultrasound”) AND (“Ultrasound” OR “Medical Ultrasound” OR “Diagnostic Ultrasound” OR “Sonography”); (“AI applications” OR “Automated Analysis” OR “Image Processing”) AND (“Transcranial Doppler” OR “TCD” OR “Cerebrovascular Ultrasound”) AND (“Ultrasound” OR “Medical Imaging”); (“Artificial Intelligence” OR “Machine Learning”) AND (“Ultrasound” OR “Medical Ultrasound” OR “Diagnostic Ultrasound”); and (“Deep Learning” OR “Convolutional Neural Networks”) AND (“Transcranial Doppler” OR “Cerebrovascular Ultrasound”) AND (“Automated Diagnosis” OR “Pattern Recognition”). Boolean operators (AND, OR) were used to refine the search strategy, ensuring comprehensive coverage of the literature discussing both current applications and future directions of AI in these imaging modalities. Additional articles were identified through a manual screening of reference lists in relevant reviews and original research papers. Only peer-reviewed articles published in English were included. In this review, a total of 12,209 articles were initially identified through a comprehensive literature search. To enhance inter-reviewer consistency, all reviewers initially screened a common set of 50 publications, followed by having a discussion on the outcomes to refine and update the screening and data extraction guidelines before commencing the full review process. A total of three reviewers, organized in pairs, independently and sequentially assessed the titles, abstracts, and, subsequently, the full texts of all records retrieved through the search strategy for potential eligibility. Discrepancies in study selection or data extraction were resolved through consensus, with input from additional reviewers when necessary. A data extraction form was collaboratively designed by two reviewers to define the variables to be collected. The reviewers independently extracted the data, compared their findings, and refined the extraction form through an ongoing, iterative process. Following the rigorous screening process based on predefined inclusion criteria, such as relevance to the topic, publication in the English language, and scientific significance, a final set of 60 articles was selected for in-depth analysis. This selection aimed to ensure the inclusion of the most pertinent and methodologically robust studies to provide a comprehensive and critical overview of the current state of research in the field. The research flow diagram is reported in Figure 1 in accordance with the PRISMA methodology [5]. Preferred Reporting Items for Systematic reviews and Meta-Analyses extension for Scoping Reviews (PRISMA-ScR) Checklist is available as Supplementary Materials [6]. Finally, no prior protocol has been registered for the development of this scoping review.

## 3. Data Provenance and Analysis

This review included 41 studies on the application of artificial intelligence (AI) in neurosonology in the diagnosis and monitoring of vascular and parenchymal brain pathologies. All studies were published between 2012 and 2025.

In terms of geographical diversity, most datasets come from North America (mainly the US) and East Asia (mainly China and South Korea). Few studies come from Europe (in particular the UK, Sweden, and Italy), and even fewer from middle-income countries. This difference in geographical coverage may limit the generalizability of some AI models developed and validated predominantly on populations in America and East Asia.

Heterogeneity in data access and geographic distribution has a potential impact on both the external validity of AI algorithms in neurosonology and the replicability of results reported in the literature. We abstracted data on article characteristics, including Year Of Publication, Sample Size, Data-Source Origin, Algorithm, Validation Strategy, and Performance Metrics. In this scoping review, a comprehensive assessment of the current diagnostic applications of artificial intelligence (AI) in Transcranial Doppler (TCD) and ultrasound was conducted, with a focus on its capacity to support clinical decision-making and optimize diagnostic workflows. This review also critically examined the principal challenges impeding the routine implementation of AI in clinical settings, including issues related to data harmonization, algorithmic transparency, and regulatory compliance. Through a synthesis of available evidence, this review aimed to underscore the transformative role of AI in enhancing the diagnostic effectiveness of TCD and ultrasound modalities. To assess the methodological quality of the included sources, the AMSTAR tool, an established and validated instrument for evaluating systematic reviews of randomized controlled trials, was employed. This tool provides an overall quality score, categorizing studies as high quality (scores ≥ 8), moderate quality (scores 4–7), or low quality (scores ≤ 3). Quality appraisal data were systematically incorporated into the data extraction form, which was initially piloted on a random subset of seven included studies, representing a range of quality scores. This process ensured the consistent application of the tool across all eligible sources.

### Data Synthesis

For data synthesis, the included studies were grouped according to the most common clinical conditions in which neurosonology is applied. These categories included the following: intracranial stenosis, occlusions, and cerebral perfusion; subarachnoid hemorrhage; microemboli detection and right-to-left shunt; monitoring in acute neurovascular care and non-invasive intracranial pressure measurement; and transcranial brain parenchyma sonography. This thematic organization facilitated a structured analysis of the diagnostic applications of neurosonology and allowed for the identification of trends, gaps, and opportunities for future research within each clinical domain.

## 4. Neurosonology: Indications and Utility

Neurosonology is a growing field that includes the use of TCD, TCCD, and transcranial sonography (TCS), finding its crucial role in the diagnosis, monitoring, and therapeutic management of neurovascular and neurodegenerative diseases.

TCD allows for the real-time monitoring of cerebral hemodynamics by measuring the blood flow velocity in basal intracranial arteries. Its application is essential for the early diagnosis of life-threatening conditions, such as subarachnoid hemorrhage (SAH)-related vasospasm, intracranial arterial stenosis, sickle cell vasculopathy, acute arterial occlusions, intracranial hypertension, and cerebral circulatory arrest [7,8]; for the assessment of the mechanisms of cerebral blood flow maintenance for cerebral autoregulation and vasomotor reactivity; and for the detection of microembolic signals in the stroke risk assessment or the intraoperative monitoring [9].

On the other hand, TCS provides structural imaging of the cerebral parenchyma, giving valuable information on neurodegenerative diseases. TCS can detect the hyperechogenicity in the substantia nigra, a hallmark sign of Parkinson’s disease, facilitating early and differential diagnoses [10]. Moreover, TCS visualizes the basal ganglia and brainstem structures, playing a pivotal role in the discrimination among Parkinsonian syndromes and other movement disorders, such as progressive supranuclear palsy and multiple system atrophy [11]. In studies on dementia, TCS has been used to detect lenticular nucleus hyperechogenicity in Creutzfeldt–Jakob disease and structural changes in the raphe nuclei in depressive illnesses in Alzheimer’s disease [12,13].

Neurosonology is also finding increasing application in neurocritical care, particularly with TCCD, which affords both hemodynamic and anatomical imaging. It is especially helpful in the monitoring of traumatic brain injury, aneurysmal subarachnoid hemorrhage, hydrocephalus, and cerebral perfusion in intensive care units [14]. The possibility of using portable devices and real-time data acquisition allows for the ease of bedside diagnosis and monitoring in emergency and critical care settings. Recent advancements like contrast-enhanced ultrasound and fusion imaging with MRI and PET have even extended their diagnostic range, rendering neurosonology a highly accurate and versatile technique for neurological assessment [15].

## 5. Transcranial Doppler and Transcranial Color-Coded Doppler: Advantages and Limitations

As mentioned above, TCD and TCCD are essential non-invasive tools for assessing cerebral diseases, but these techniques show advantages and limitations. The advantages of TCD include portability, cost-effectiveness, and real-time data acquisition. However, its effectiveness depends on operator expertise and may be compromised by inadequate acoustic windows, especially in elderly patients with a higher prevalence in females. Conversely, TCCD integrates Doppler ultrasound with B-mode, allowing the direct visualization of the intracranial vascular system. This combination enhances the accuracy of flow velocity measurements and aids in identifying vascular anomalies such as vasospasm [16]. The ability of TCCD to provide angle-corrected velocities and detailed anatomical insights is counterbalanced by the need for advanced equipment and greater operator expertise. Additionally, like TCD, TCCD effectiveness may be limited by suboptimal acoustic windows [17]. Both techniques are valuable in neurocritical care and cerebrovascular evaluations. TCD offers a simple and portable option for rapid assessments, whereas TCCD provides comprehensive anatomical and hemodynamic information but at the expense of increased complexity and resource utilization [18].

TCD and TCCD are also utilized in primary stroke prevention for conditions such as intracranial stenosis, asymptomatic carotid stenosis, and patent foramen ovale (PFO). Intracranial stenoses represent one of the most frequent causes of stroke worldwide and are associated with a poor prognosis [19], allowing for the stratification of the patients into classes of risk for acute cerebrovascular events and recurrent stroke, in particular in the populations with a higher prevalence (Asian, African, and Hispanic populations) [20]. Indeed, the diagnostic peak systolic velocity (PSV) thresholds for stenosis greater than 50% in the middle cerebral artery, validated by angiographic studies, are 200 cm/s for mild and moderate and 280 cm/s for moderate and severe, and the risk of stroke is proportional to the severity of the stenosis [21]. Comparative studies between TCCD and the recently developed 3D, high-resolution, black-blood MR sequence show that TCCD has an 80% accuracy in identifying and discerning the etiological diagnosis of intracranial stenosis [22], providing a valid tool for the non-invasive preventive diagnosis and the early indication of dual antiplatelet therapy. TCCD can identify arterial stenoses and quantify their severity, whereas TCD can permit the detection and counting of intracranial microembolic arterial signals (HITSs—high-intensity transient signals) originating from carotid, cardiac, or aortic embolic sources [23]. Furthermore, with contrast-enhanced ultrasound, TCD can identify interatrial shunts. Studies such as NASCET, ECST, and VACS [24] have demonstrated a net benefit of surgical intervention in symptomatic patients with carotid stenosis greater than 70%. In 2010, the ACES study showed that the detection of HITSs via TCD allows for the identification of a subgroup of patients with asymptomatic carotid stenosis who could benefit from surgical treatment [25]. In 2017, this selection criterion was included in the ESC guidelines (grade IIa) for asymptomatic carotid stenoses between 60% and 69% [26,27]. Moreover, the presence of microembolic signals detected by TCD after contrast administration indicates the presence of a shunt, and their number defines its magnitude with an accuracy comparable to transesophageal echocardiography [28].

However, despite the valuable role in cerebrovascular assessment of both techniques, Transcranial ultrasonography has several limitations that may affect its diagnostic reliability and clinical applicability. One of the main limitations lies in the requirement of a suitable acoustic window, which is not observed in nearly 10–20% of the patients, especially in elderly patients, with a higher prevalence in women and those with hyperostosis of the temporal bone [29]. This limitation can lead to poor insonation of intracranial arteries, which consequently lowers the precision of velocity measurements along with anatomical delineation clarity. Another challenge relates to operator dependence because TCCD quality and the reproducibility of results are closely linked to the skill of the sonographer. Proper probe positioning, angling, and spectral waveform analysis require extensive practice, yet operator variability remains a cause for concern [30]. In addition, TCCD has poorer spatial resolution compared to other neuroimaging modalities, like computed tomography angiography (CTA) or magnetic resonance angiography (MRA), which provide greater structural detail about vessel morphology and collateral circulation [31]. In addition, although TCCD can measure intracranial stenosis and hemodynamic changes, it is weaker in detecting distal small vessel disease and some embolic pathologies. Its measurement of the degree of stenosis is dependent on flow velocity thresholds, which are affected by systemic hemodynamic change, cardiac output, or collateral circulation [32]. Finally, TCCD requires advanced equipment and technical expertise for its widespread application in non-specialized centers. Despite these limitations, TCCD is an excellent tool for the non-invasive study of cerebrovascular disease, particularly for the real-time monitoring of cerebral hemodynamics and the guidance of therapeutic interventions. In this setting, AI could play a crucial role in overcoming the limitations of TCD and TCCD and exploiting and implementing the potential of these diagnostic tools.

## 6. Generality of Artificial Intelligence

AI can be broadly defined as the deployment of technological systems designed to replicate human cognitive functions, encompassing processes such as learning, reasoning, and problem-solving [33]. This concept has formed the foundation for the evolution of computational tools capable of simulating thought and analytical decision-making. The first AI-powered medical technology received approval from the United States Food and Drug Administration (FDA) in April 2018 [34], specifically for an ophthalmic system used to detect diabetic retinopathy. Following this milestone, various AI-driven healthcare applications emerged, each addressing different clinical needs. The healthcare sector has increasingly integrated AI methodologies, utilizing sophisticated algorithms to assess risk factors, optimize treatment strategies, and forecast patient outcomes based on extensive multimodal datasets [34]. AI comprises several subfields, including machine learning (ML), deep learning (DL), and convolutional neural networks (CNNs). ML, as a subset of AI, encompasses a range of learning techniques, each designed to address distinct types of challenges, from automating repetitive tasks to performing predictive analyses. Unlike traditional programming, ML algorithms adapt and refine their decision-making abilities based on data exposure, eliminating the necessity for explicit programming rules [35]. The ML process typically involves three main phases: training, testing, and validation. Algorithms within this framework are classified into three primary categories: supervised learning, unsupervised learning, and reinforcement learning. Supervised learning, the most frequently applied technique in diagnostic imaging, entails training models on labeled datasets. This approach enables algorithms to recognize patterns and apply learned knowledge to predict outcomes for previously unseen data. In medical diagnostics, for instance, an AI system may be trained on datasets with known outcomes to develop predictive capabilities for new, unlabeled cases [36]. Conversely, unsupervised learning operates without predefined labels, instead identifying inherent structures or correlations within the data. This method is valuable for clustering and categorizing medical information based on shared characteristics, such as laboratory results, clinical symptoms, patient demographics, or genetic markers. Reinforcement learning differs from these approaches by employing a trial-and-error mechanism, where an algorithm iteratively refines its decision-making process through feedback received in the form of rewards or penalties [37]. This allows the system to autonomously improve its performance over time while adhering to predefined operational constraints. ML can also be categorized based on whether it relies on predefined features. Some models depend on domain-specific expertise to identify relevant attributes, while others extract patterns autonomously from raw data, optimizing their performance without manual intervention [38]. This latter approach, known as DL, has demonstrated exceptional efficacy across various domains, including image classification, natural language processing, and speech recognition. DL, a specialized branch of ML, employs artificial neural networks (ANNs) structured in multiple layers, inspired by the biological neural networks of the human brain [39]. This layered architecture allows DL systems to process and interpret vast amounts of heterogeneous data, including text, audio, and visual content. The primary objective of DL is to emulate human neural processing by enabling systems to learn directly from raw data inputs and to generate meaningful insights without predefined rules [40]. DL architectures typically consist of multiple neural network layers, where each subsequent layer refines and abstracts features extracted from the preceding layer. This hierarchical structure enhances the model’s ability to capture complex patterns and relationships within large datasets, leading to groundbreaking advancements in areas such as automated speech recognition, image analysis, and medical diagnostics [41]. The rapid progress of DL has been facilitated by the availability of extensive datasets, enhanced computational power, and efficient training algorithms such as backpropagation. Consequently, DL has revolutionized numerous applications, from autonomous vehicle navigation and biometric recognition to pharmaceutical research and clinical decision support. The depth and complexity of an ANN influence its analytical capabilities, with deeper architectures capable of more sophisticated data representations. Notable DL architectures include CNNs, recurrent neural networks (RNNs), variational autoencoders, and generative adversarial networks (GANs). Despite the advanced capabilities of DL, several limitations must be considered. A significant challenge is the necessity for large and diverse training datasets to achieve reliable and reproducible results [42]. Additionally, model accuracy is contingent on data quality, necessitating rigorous preprocessing and validation. Furthermore, human oversight remains crucial, as clinicians must interpret and contextualize AI-generated insights to ensure alignment with established medical knowledge and clinical expertise [43]. This is particularly relevant in fields such as radiology, neurology, and dermatology, where AI is increasingly employed to enhance diagnostic precision through automated image analysis. Among the various applications of AI in medicine, image-based diagnostics remains one of the most extensively researched and implemented areas.

## 7. Intracranial Stenosis, Occlusions, and Cerebral Perfusion

Intracranial atherosclerotic stenosis (ICAS) is one of the recently emerging causes of ischemic stroke [44,45], responsible for 8 to 37% of acute cerebrovascular events [46]. The term ICAS is comprehensive of the presence of atherosclerotic obstruction on the middle cerebral artery (MCA), the intracranial portion of the internal carotid artery, the vertebrobasilar artery, and the posterior and anterior cerebral arteries. The MCA is the most susceptible artery to develop atherosclerotic lesions, increasing the risk and the recurrence of transient ischemic attacks (TIAs) and strokes [47]. The imaging techniques currently used to directly identify ICAS are the MRA and CTA. Still, these tools have some limitations, such as the costs, invasiveness, and radiation exposure. TCD and TCCD are two non-invasive, cheap, quick, and easily reproducible techniques for the evaluation of ICAS [48,49]. Given the small size of the intracranial arteries, TCD and TCCD cannot directly visualize the intracranial atheroma but can identify the alterations due to the stenosis; indeed, following the Poiseuille law, ICAS causes a variation in the blood flow velocity at the point of the stenosis and downstream, detectable by the use of low-frequency ultrasound (<2 MHz) probes. Thanks to the modifications of the waveform and spectrogram and the variations in the flow-dependent variables (PSV, end-diastolic velocity (EDV), minimum diastolic velocity (MDV), resistance index (RI), and pulsatility index (PI) [50,51,52,53,54,55,56]), TCD and TCCD may help in the diagnosis and stratification of ICAS. The analysis of the PSV values allows for the classification of the MCA obstruction into mild (140 cm/s ≤ PSV < 180 cm/s), moderate (180 cm/s ≤ PSV < 220 cm/s), and severe (PSV ≥ 220 cm/s), with an accuracy comparable to CTA imaging [57]. The accuracy of the TCD/TCCD is greater than the degree of stenosis, achieving a rate of 89% in the case of a MCA stenosis of more than 50% [58]. However, some limitations, such as the possibility of poor ultrasound transmission due to the thickness of the temporal bone or the need for expert operators requiring strict training [59], can affect the precision of the exam. To overcome these difficulties, fully automated methods for easily identifying TCD variables were proposed several years ago. Recently, the application of ML and NLP techniques is gaining ground more and more in the automatic conversion of free-text input into structured data, identifying ICAS in a rapid and automated way [60].

### 7.1. Application of AI to TCD for ICAS Diagnosis

AI has started to be applied to ameliorate TCD image analysis and eliminate potential mistakes by manually analyzing the features for the intracranial hemodynamic evaluation. At first, two different methods, the adaptive autoregressive moving average (A-Arma) and the fast Fourier transform (FFT) methods, were used to examine the spectrum signals, with the first showing superiority over the latter [61,62]. Later, Serhatlioğlu et al. used FFT to extract TCD signals, then sent them and classified them by an ANN [63]. In 2008, chaotic invariant features from TCD signals were classified using two different neuro-fuzzy classifiers, called ANFIS and NEFCLASS, to identify, among the 82 patients recruited, different signals based on the blood alterations (aneurysm, cerebral hemorrhage, cerebral edema, brain tumor). From these results, Uğuz et al. conducted a series of studies in a period ranging from 2008 to 2012, finally evaluating the efficacy of the methods using the support vector machine (SVM) classifier to find an accurate extraction and classification system [64,65]. Subsequently, Seera et al. resorted to an RNN algorithm to evaluate the cerebral Willis circle in patients with acute ischemic stroke, with an accuracy ranging from 70 to 85% [66]. However, few studies have yet been conducted on applying DL to TCD images to identify ICAS. In 2011, Myrden et al. [67] evaluated the application of a brain–computer interface-based method useful for detecting the variations in the cerebral blood velocities on TCD based on mental activity, resulting in an accuracy of > 80%. This system was later applied in other studies [68,69], confirming the feasibility of creating connections between the human brain and external devices to detect changes in the cerebral vessels. In one study of Hsu et al. [70], 8211 patients were enrolled and underwent carotid color Doppler ultrasound and TCD to collect extra-intracranial features. The activation of collateral circles leads to hemodynamic changes useful for diagnosing intracranial stenosis. Flow velocity (FV), PSV, and EDV were the parameters used to detect ICAS indirectly. In the case of intracranial ICA stenosis, there was a reduction in FV, which then appeared in the extracranial ICA and MCA, while the increase in FV and also the increase in PSV and EDV were primarily seen in EICA stenosis than in ICA and MCA stenosis. The sensitivity of the measurements was high (90%, 80–90%, and 70–80%, respectively, for FV, PSV, and EDV), but the scarce specificity (from 30 to 60%) made these evaluations not appropriate for predicting intracranial stenosis. Thus, to optimize the accuracy, an SVM algorithm was created to easily and quickly detect these changes in the hemodynamics. A concurrent angiographic study confirmed the results. In this study, the use of ML tools allowed for an increase in the accuracy and sensitivity of traditional techniques, achieving values of up to 20% and 45%, respectively. Hence, applying AI to color Doppler and TC ultrasounds may help to discriminate patients with significant intracranial stenosis who deserve to undergo other invasive studies.

In 2021, O’Brien et al. proposed the NovaGuide system for the evaluation of the mean cerebral flow velocity (mCBFV) and signal quality assessment with an accuracy comparable with expert revelations but with the possibility of faster acquisition [71]. In another study by Mei et al. [72], 203 patients without MCA stenosis and 73 patients with MCA stenosis were enrolled and underwent a TCD study; the evaluated parameters were the PSV and the mean flow velocity (MFV), which were higher in the case of stenosis. The accuracy of ICAS detection with Transcranial Doppler was similar to that of angiographic studies, with a sensitivity, specificity, and area under the curve (AUC) of 0.80, 0.83, and 0.81, respectively. The application of a CNN model from TCD images showed a stackable accuracy (the sensitivity, specificity, and AUC of the CNNs in the test set were 0.84, 0.86, and 0.80, respectively), providing a potential tool to facilitate diagnosis. In 2022, an ML-based clinical decision support system used the TCD parameters (PSV, EDV, and MFV) of the ACA, MCA, PCA, VA, and BA collected from Digital Imaging and Communications in Medicine (DICOM), with an overall accuracy ranging from 67 to 86% [73]. In this study, PSV, EDV, blood flow volumes, vascular diameters of the vessels, and the PI or RI values were the variables obtained from all of the arteries. The results showed that M1 distal/proximal PSV, M1 distal/proximal PI, M2 PSV, and M2 PI were good predictors of MCA stenosis, while PSV and PI values predicted VA or BA stenosis. In a recent study in October 2024 [74], 1729 hospitalized and outpatient patients were enrolled and tested for intracranial MCA stenosis through a TCD study. Images from transcranial studies were then analyzed by a machine learning algorithm called VGG16. In VGG16, the image is sent to the five convolutional blocks, the “Feature Extraction Layer,” and three fully connected layers. This DL technique appeared to be able to ease the detection of the ICAS, with an accuracy, specificity, and sensitivity of 85.67 ± 0.43(%), 87.73 ± 1.47(%), and 83.60 ± 1.60(%), respectively. AI-applied TCD allows for the application of more than one hundred features for diagnosing ICAS, unlike the restricted features used by specialized neurosonologists. Thus, AI may be a promising tool for the future use of the TCD in stroke screening and evaluation.

One of the limitations of the above studies is that ML techniques have a good accuracy related to detecting the grade of stenosis, but show weakness in the case of mild stenosis. Several authors proposed a classification DL algorithm to overcome this limitation based on the variation in mCBFV waveforms. Finally, Nisha et al. [75] constructed two Self-Organizing Operational Neural Network-based novel 1D classifications to achieve a binary classification of MCA stenosis with an accuracy, specificity, and sensitivity of more than 95%. This model was built from previous studies using a Learning Vector Quantization Neural Network to classify ICA stenosis [76,77]. The main characteristics of studies applying AI to TCD in ICAS are reported in Table 1.

### 7.2. Transcranial Doppler for the Diagnosis of MCA Occlusion

TCD can also be used for evaluating the perfusion state of the cerebral tissue after MCA-occlusion-induced stroke. TCD has shown a high diagnostic accuracy comparable with the CTA study in detecting MCA occlusion. Guan et al. recruited 128 patients with MCA occlusion, and 68% underwent both CTA and TCD studies, with a mean time interval of less than half an hour between the examinations. TCD showed a sensitivity of 100% and specificity of 98.9%, suggesting the possible application of the method for the early diagnosis of MCA occlusion [78]. To improve the accuracy of the diagnosis, Kilic proposed a novel technique using the TCCD curves during the concomitant injection of the echo-contrast agent to evaluate the cerebral perfusion state [79]. This model, named mobile brain perfusion ultrasound, was applied to a 46-year-old patient with left hemispheric ischemia and angiographically CT-diagnosed MCA occlusion. Brain perfusion ultrasound was performed in this patient before the mechanical thrombectomy and showed a significant delay (6.6 s) in left hemispheric perfusion compared to the traditional TCCD. However, the use of BPU remains valid only in the prehospital setting [80,81].

In the case of MCA occlusion, leptomeningeal anastomoses, which connect the MCA to the posterior and distal anterior cerebral arteries (PCA and ACA2, respectively), are activated and ensure tissue perfusion [82]. The presence of this collateral anastomosis allows for improvement in the perfusion impairment, enhancing the future prognosis. The activation of collateral circulation and the diversion of cerebral flow can lead to changes in the velocity waveforms as a consequence of the variation in vascular resistances, which is related to the PI. Because of the need to ensure a constant blood supply, in the case of MCA stroke, the PIs of the ipsilateral ACA and PCA are reduced, while non-significant modifications occur on the contralateral arteries. Thus, the evaluation of the PI of homolateral and contralateral ACA1-2 and PCA is related to the capacity of the leptomeningeal anastomoses to provide cerebral flow redistribution after acute stroke. Benemerito et al. [83] identified modern biomarkers correlated with the lack of cerebral perfusion after acute ischemic events to select patients needing quick intervention. Their study evaluated the correlation between the PI and the product of pulsatility (PPI), which comes from the combinations of PIs of different arteries with distal perfusion. A negative correlation was found. The cut-off values for the identification of a lack of perfusion were 1.6 for the ipsilateral ACA-PI and 9.3 for the PPI. The other biomarkers showed the same behaviors. ML-based 1D models were proposed to obtain a scatter plot for the identification of patients with poor prognoses. Flow diversion resulted in an increase in the FV in the ipsilateral ACA and/or ACP compared to the contralateral, which represents a compensation mechanism for the interruption of the blood flow in the case of MCA occlusion. In their study, Kim et al. [84] observed that the detection of these TCD variations corresponds to the activation of the leptomeningeal anastomosis. In this study, 51 patients with ischemic stroke due to MCA occlusion were recruited; the obtained results showed that an increase in the flow velocity of more than 30% in the ipsilateral artery compared to the contralateral was associated with the LMAs opening, as demonstrated by the concomitant angiographical studies. Flow velocity increased predominantly in the ACA than in the ACP artery, presumably due to the larger number of collaterals with the MCA.

The importance of the early detection of flow velocity comes from the evidence that the absence of flow velocity after MCA occlusion is related to a worse outcome within 24 h of occlusion because of the lack of perfusion of the distal MCA, leading to permanent cerebral damage [85]. Considering the consequences of MCA occlusion, early diagnosis represents a diagnostic goal that needs to be accomplished. In these terms, AI may be a useful tool to improve diagnostic sensitivity through the application of automated algorithms to the TCD study.

However, even if some studies have been carried out on the AI-based algorithm using CTA imaging for the detection of MCA occlusion [86,87,88], no data are available on the possible application of AI for building new TCD-based models for improving diagnosis. Many efforts may be made to gain new pieces of knowledge in the scientific community.

### 7.3. Maintaining Cerebral Perfusion: Transcranial Doppler in Cerebral Autoregulation

Cerebral autoregulation is one of the mechanisms activated in the case of ICAS. As a consequence of various stimuli, cerebral vessels dilate to maintain the cerebral perfusion, ensuring the cerebrovascular reserve. The ability of the cerebral arteries to change their diameters to guarantee the balance between the metabolic demand and vascular supply is called “cerebrovascular reactivity,” and its impairment increases the risk of cerebrovascular events [89,90]. The gold standard for the evaluation of the maintenance of cerebral autoregulation, the TCD study, was used to estimate the variations in cerebral resistance and flow. The autoregulatory function is derived from the relationship between the M waves (spontaneous oscillation of CBF) of arterial blood pressure, which represent the input function, and FV, representing the output function. B waves are cerebral oscillations of slower frequencies [91]. Several studies have demonstrated that variations in blood pressure imply a cerebral blood flow change [92,93,94]. While several studies have been carried out on the variations in the cerebral blood flow change as a consequence of carotid stenosis [95], few data are available on cerebral autoregulation in the case of MCA stenosis. In 2003, twenty-two patients with TCD-detected M1 stenosis were recruited and underwent a TCD study to evaluate the variations in cerebral blood flow. The study revealed a reduction in the M wave phases and an increase in the B wave phase in the case of moderate–severe stenosis degree in comparison to the control group, advising of an impairment in the arteriolar function related to severe intracranial stenosis [96].

Later, Wang et al. evaluated dynamic cerebral autoregulation in sixty-five asymptomatic patients with unilateral MCA stenosis, divided into subgroups based on the degree of the stenosis [94]. The study showed that the phase difference [97] and the gain values were significantly lower only in the group with severe stenosis ipsilaterally to the obstruction, suggesting an impairment of the dynamic cerebral autoregulation in these cases. AI has been applied in the study of cerebral autoregulation. A time-lagged recurrent neural network was employed to evaluate the relationship between arterial blood pressure and changes in cerebral blood flow velocity in sixteen healthy subjects [98]. A device was used to measure radial arterial pressure, and continuous TCD monitoring was used to detect variations in cerebral flow velocity. The automated model showed good performance compared to another time-domain linear method, laying the basis for the construction of other models to examine other physiological tests of CA, such as the Valsalva maneuver, hand-grip test, head-up tilt, or the response to CO_2_ inhalation, but also to predict the prognosis in patients with acute ischemic stroke, evaluating the capability of the cerebral arteries to regulate the cerebral blood flow and guarantee parenchymal perfusion despite the obstruction. The above data may lay the foundation for future studies to ensure the earlier diagnosis and better risk class stratification of patients with intracranial stenosis.

## 8. Subarachnoid Hemorrhage

SAH is a life-threatening condition whose incidence is 6-9/100.000, and its prevalence is 3.2% in the general population [99]. Despite the improvement of surgical techniques, this condition is still burdened by high mortality and residual disability [100,101]. The primary cause of SAH is intracranial aneurysmal rupture, often causing massive intracranial parenchymal and ventricular hemorrhage [102]. One of the main complications of SAH is vasospasm-induced delayed ischemia, with an incidence of up to 40%. Cerebral vasospasm leads to neurological deterioration, negatively affecting the prognosis of patients with SAH. For this reason, early diagnosis is important to apply prevention and therapeutic strategies to minimize cerebral damage. Thus, patients with SAH are continuously monitored in the intensive care unit (ICU) wards. In the case of SAH, the released hemoglobin induces the entry of calcium into the smooth cells of the vascular layers, activating the calcium/calmodulin-dependent myosin light-chain kinase responsible for the actin and myosin cross-linkage and the consequent contraction. However, chronic vasospasm, occurring days after acute bleeding, is less sensitive to calcium levels, depending on other pathways such as the contractile proteins protein kinase C, Rho kinase, and protein tyrosine kinase signals; this is the reason why in the chronic phase, calcium-channel inhibitors lack efficacy in the treatment of vasospasm and, on the other hand, is the reason for the importance of pharmacological prevention [103,104]. The scarce reversibility of the vasospasm also derives from the irreversible vascular damage induced by methemoglobin and superoxide anion radical production, leading to lipid peroxidation and reduced expression of vasodilator agents (such as NO) or the increased expression of endothelin [105,106], and on the other hand, the vascular inflammation induced by inflammatory cytokines [107]. The subacute onset of headache and behavioral changes are the usual symptoms of vasospasm, while rapid neurological deterioration is less frequent [108]. If not treated, the condition evolves till the appearance of focal neurological signs occurs, depending on the involved artery: hemiparesis and aphasia are typical of the MCA involvement, while ACA vasospasm leads to leg weakness, confusion, and language deficit.

### New Boundaries in Transcranial Doppler Applications: Artificial Intelligence in the Diagnosis and Monitoring of Vasospasms

The gold standard for the diagnosis of SAH is non-contrast CT. However, the identification of the vascular branch responsible for the hemorrhage, as well as the visualization of the change in the size of the vessels as a consequence of the vasospasm, requires the use of more complicated methods, such as digital subtraction angiography, high-definition computed tomographic angiography, and magnetic resonance imaging. These techniques remain the most used, even if they are expensive, involve exposure to ionizing radiation, and are less reproducible for the monitoring of patients [109]. Thus, the mCBFV increase is an indirect index of vasospasm, more easily evaluable with TCD [110], which represents a non-invasive, reproducible, and safe tool for the early diagnosis of vasospasm. Continuous TCD monitoring may be performed at the bedside to record the variations in the CBFV typical of the vasospasm. The FV increase related to the vessel diameter reduction relies on the degree of vasospasm. Generally, an increase in the CBFV more than 50 cm/s from the basal value within 24 h is a strong predictor of vasospasm [111], but an MFV > 120 cm/s indicates vasospasm whose severity is higher if the values exceed 200 cm/s [112]. The evaluation of the “Lindegaard ratio,” consisting of the ratio between the velocity in MCA and the velocity in ipsilateral ICA, allows us to distinguish vasospasm from hyperemia: a ratio value of more than 3 is indicative of vasospasm [113]. In the case of intermediate values, the combination of TCD with other maneuvers, such as the compression of the ipsilateral carotid artery, or techniques, such as the cerebral blood flow measurement, may help to increase the accuracy in the diagnosis of vasospasm [114,115]. The importance of the early diagnosis is that the variations in the cerebral velocities shown at the TCD precede the clinical manifestations by at least 24–48 h, allowing preventive measures to limit the cerebral damage. However, given the irregularity of the lesions, the different types of hemorrhage, and the possibility of misdiagnosis due to the possible overlap with the thickened membranes of the small spaces, the manual labeling of the images may be challenging [116,117]. For vasospasm detection, TCD monitoring can be hard because of some difficulties, such as the scarce insonation of the cerebral arteries, the need to maintain the correct position of the probe during the recording, and the need for trained experts. For this reason, AI-based algorithms have been constructed to aid in the diagnosis, reduce morbidity and mortality, and improve the follow-up. In 2018, Elzaafarany and colleagues [118] constructed a model for the automated detection of vasospasm based on TCD signals. The model was made on mathematical and audio features to identify the cerebral vessels and hemodynamic parameters. Patients with vasospasm were differentiated from the control group based on the MFV (more than 120 cm/s) and the intracranial-to-extracranial MFV ratio (more than 3). The features were used to build a pattern recognition model that was able to compress a high volume of data to improve the accuracy of the classifier. The model could predict cerebral vasospasm less than 9 s early and showed an accuracy of 89.17% for both groups; the errors were overtaken thanks to the application of time-averaging correction. In the same period, another study [119] proposed an automated algorithm for cerebral vasospasm detection using sequences of 3–15 s TCD audio signals: the model showed a sensitivity and a specificity of 78% and 91%, respectively, proposing the possibility of developing an automatic continuous “vasospasm monitor”. In 2020, Esmaeeli et al. [120] evaluated the application of the Lucid™ M1 Transcranial Doppler Ultrasound System^®^ and NeuralBot™ System, a robotically assisted ultrasound system approved by the FDA for the measurement of the cerebral blood flow velocity, eliminating the operator dependence and variability in two patients with severe SAH. The first patient was a 47-year-old man with a severe SAH due to the rupture of a 4 mm anterior communicating artery aneurysm; the second one was a 60-year-old man with an SAH with intraventricular hemorrhage and with an anterior communicating artery aneurysm. In both cases, the manual and robotic TCD showed stackable values of increased CBF velocities in the MCA but not in the ACA, without significant differences in the timing of the measurements. The Lucid Robotic System combines TCD images, robotic wands, and ML models to realize the best visualization and insonation of the cerebral arteries without humans having to resort to entering them. However, the model cannot provide the insonation of the posterior arteries, and its only use is restricted to the MCA waveforms with a high accuracy compared with the manual TCD and angiographic studies. A retrospective study [121] was carried out at the Westchester Medical Center and was based on a database of patients with SAH. Patients underwent a CTA study 4, 7, and 10–12 days after the SAH to evaluate the MCA vasospasm and were recruited for the study. They were subjected to TCD monitoring on the same days as the CTA studies, and images were collected using the NovaGuide System (NovaGuide Intelligent Ultrasound, NovaSignal Corp., Los Angeles, CA, USA). In the literature, vasospasm occurs when CBFV is > 86 cm/s, with a mild, moderate, or severe degree for values < 120, from 120 to 200 cm/s, and >200 cm/s, respectively [122,123]. In the above study [121], the application of a threshold value corresponding to the maximum CBFv (>120 cm/s) has a result comparable to the CTA in the vasospasm diagnosis, with a sensitivity of 83%, a negative predictive value of 90%, and positive likelihood ratio of 8.75. Recently, another study has confirmed the increase in the application of AI in SAH diagnosis. A DL method regarding a convolutional layer-based neural network was realized from 1727 Doppler wave files obtained from 19 patients with SAH; the algorithm was able to extract parameters such as the PSV, EDV, and MFV to classify the patients (normal, cerebral vasospasm, and hyperemia). The model showed an accuracy and sensitivity of 90%, supporting the commitment to introduce AI-based techniques in the diagnostic procedure of SAH [124]. In a retrospective study of the 10-year dataset from 500 patients with angiographically verified aneurysmal SAH surviving at least 7 days from admission who underwent clinical and TCD monitoring, data were extracted for building a valuable ML model to stratify patients into classes of risk for the development of vasospasm to optimize the care of patients [125]. Finally, a recent meta-analysis has collected data from eight studies that used ML algorithms applied in TCD to detect DCI after SAH. The result confirmed the potential feasibility of using AI in predicting vasospasm in patients with SAH, with a pooled sensitivity of 0.79, a specificity of 0.78, and an AUC of 0.85 [126]. The above studies have shown the feasibility of introducing AI-based algorithms for the early diagnosis of cerebral vasospasm to overcome the difficulties in TCD application, depending on the requirement of having well-trained operators and the time needed to be expended for monitoring. Introducing these new technologies in clinical practice opens up new horizons for the improvement of the management of patients with SAH, aiming for a reduction in clinical costs and long-term disability and mortality. The main characteristics of studies applying AI to TCD in SAH are reported in Table 2.

## 9. Microemboli Detection and Right–Left Shunt

Cerebral embolism is one of the leading causes of ischemic stroke [127]. Several conditions are considered as possible sources of cerebral emboli: First of all is atrial fibrillation, but other causes are also correlated with cerebral embolism. In 2014, the term “embolic stroke of undetermined source (ESUS)” appeared for the first time, indicating non-lacunar strokes not associated with proximal carotid stenosis or a cardioembolic source. The more frequent causes of ESUS are hidden atrial fibrillation (in 44% of cases), emboli from unstable carotid plaques, and PFO [128]; other conditions, although possible, are very rare. TCD is the gold standard for identifying cerebral embolic events, thanks to its capability of registering HITSs. HITSs are high-intensity transitory signals that appear as bright, high-amplitude spikes, typically ≥3 dB higher than the background blood flow signal, lasting <300 milliseconds. They are identified as “squicking noises” consisting of the increased intensity of the power Doppler signal on the background spectrum and representing the transfer of microemboli from systemic to cerebral circulation [129]. However, the intensity of the HITS is comparable to the artifact sounds. For this reason, a strict formation of expert operators needs to be achieved to avoid pitfalls. Therefore, this requirement limits the use of this method to specialized centers, restricting the possible diagnostic resources. The application of automated tools can overcome these limitations: the use of neural networks applied to the TCD study may help to avoid mistakes and improve the accuracy of the diagnosis. Studies about the application of automated algorithms found their roots several years ago. In 1999, Kémeny et al. [130] recruited 11 patients with cerebral embolism from cardiac or arterial sources and 11 healthy controls. All the individuals underwent TCD registration for 60 min with the neural network software EMBotec V5.1. One was applied to evaluate the capacity to discriminate the “real” microembolic signals from the artifacts. As a result, in the group of patients, 282 signals were recorded, but 122 of these were artifacts, while 58 real microembolic signs were not detected. On the contrary, in the control group, among the 1342 provoked artifacts, 216 signals were labeled as HITSs. This algorithm showed a sensitivity of 73.4% and a positive predictive value of 56.7%, with higher accuracy in the subgroup of patients with microemboli from prosthetic cardiac valves. The limitation of the EMBotec is that the method cannot reject all the artifact signals over 10 dB, requiring a good signal-to-noise ratio and an embolic signal between 1 and 10 dB. A previous study [131] had yet to evaluate the application of the same neural network in detecting cerebral microembolism in patients with high-grade carotid stenosis. Unfortunately, the device showed poor sensitivity (62%). This network was compared with two other settings (RB11 showing a sensitivity of 70% and the Pioneer 44%), suggesting the need to resort to expert observers to improve the method’s accuracy. In 2006, a system derived from a combination of a programmable dual-channel/multigate TCD unit and a digital signal processor was built: the unit, Candidate Embolic Event Detection, registered the scattered signals using a blackboard of 164 rules. The system could register some information, such as the direction of the signals, the measured embolus-to-blood ratio higher than 7 dB, and the unique distribution of the gates, to distinguish between the HITS and the artifact. Preliminary in vitro experiments showed a sensitivity of 93% and a specificity of 99%, values stackable compared to in vivo results [132]. Recently, a conventional 2D CNN model was applied to TCD to extract time–frequency representations of the microembolic signs to classify cerebral emboli. The model was made of four convolutional blocks which had 2D convolutional filters. The model gave two complementary representations of the HITS based on the temporal (raw signal) and spectral (spectrogram) information. These two representations were then fused by the automated model to pursue the final classification score to insert the microembolic signs into the specific classes [133]. The data from the literature reported below discuss the application of artificial intelligence to transcranial monitoring in different potential sources of cerebral embolism and ESUS.

### 9.1. Patent Foramen Ovale

PFO is one of the most frequent cardioembolic sources in ESUS. This anatomical condition has a prevalence of 25% in the general population and up to 40% in patients with ischemic stroke, representing one of the significant etiologies of cryptogenic stroke [134]. The right-to-left (RTL) shunt is responsible for the paradoxical transition of thrombi from the lower limb to the arterial circulation and the cerebral arteries, leading to ischemic events. Improving the diagnostic approach in the detection of PFO has a considerable impact on the prognostic stratification of patients with ischemic stroke. TCD is a more sensible, non-invasive, and less expensive method for detecting interatrial defects. The presence of the shunt is confirmed by the detection of microembolic signals on the MCA [135,136] after administering a solution of agitated hydrosaline mixed with air into the brachial vein. The severity of the shunt is correlated with the number of HITSs [137]. The sensitivity of the TCD ranges from 68 to 100% in detecting microembolic signs, but one of the limitations is the impossibility of identifying the source of the embolism. Thus, the application of automated TCD may help to improve the diagnostic approach for the detection of PFO, overcoming the difficulty of the invasive transesophageal echocardiogram (TEE) and improving the diagnostic approach in cerebrovascular disease. A large number of studies applying artificial intelligence to the detection of cerebral microembolism concern the presence of PFO. Robot-assisted TCD (raTCD) has been applied by Rubin and colleagues in patients with presumed RTL shunt-related stroke or TIA [138]. The study was conducted by coordinators with no expertise in TCD and enrolled 129 patients who underwent the raTCD study. The method was compared to the TEE and showed a higher accuracy in diagnosing the presence of PFO: in fact, raTCD showed a sensitivity of 64% in comparison with the 20% of the TEE. Moreover, the raTCD detected a large RTL shunt in 27% of cases, picking out patients who most likely needed the PFO closure. This protocol has some limitations, such as the impossibility of detecting some anatomical characteristics, such as septal aneurysm, which can lead to the worsening of the shunt, leading to a high risk of ischemic events. The recent BUBL study confirmed the superiority of the automated TCD-based algorithm over the TEE. RaTCD was able to identify the presence of right-to-left shunt with a rate three times higher than the TEE, with an accuracy much higher in the case of large-dimension shunts [139]. A retrospective cohort study associated the raTCD Spencer Logarithmic Scale with the Risk of Paradoxical Embolism to identify patients at risk of PFO [140]. Among the 212 patients with a diagnosis of ischemic stroke/TIA, 55.4% had a Spencer Logarithmic Scale of 1–2 and were identified as “unlikely to have PFO”; 30.4% had a Spencer Logarithmic Scale > 5 and were classified as “Probable PFO”; and the intermediate value of the Spencer Logarithmic Scale (3–5) was classified as “Possible PFO”. The system was called the Modified Screening of PFO-Associated Stroke Causal Likelihood. It may be introduced in clinical practice to stratify PFO risk and select those who need further evaluation. Recently, Keunen et al. built a novel algorithm that distinguishes gas embolic signals from artifacts in patients with PFO-associated microembolism. The algorithm was based on a pattern recognition model and used three parameters and the intensity and zero-crossing index to quantify these HITS dynamics and the velocity. Emboli have a high velocity and a low zero-crossing index value, contrary to artifacts. The algorithm detected shower and curtain signals, occurring in about 44% of the population in the exam, and associated with the severity of the shunt. However, the method did not identify almost 25% of the curtains because of an intensity lower than 3 dB, which is the indicated cut-off to discriminate the HITSs from the artifacts. In conclusion, the system showed a sensitivity and specificity of 96.4% for the microembolic gaseous signals with an intensity above the value of 3 dB, but expert operators were still needed to avoid misdiagnosis in the case of less intensive signals [141]. A retrospective project recruiting 148 patients with ischemic stroke admitted between February 2021 and February 2023 at the CHI Memorial Hospital in Chattanooga found that raTCD had a higher accuracy than the transthoracic echocardiogram (TTE) in detecting the RTL shunt. raTCD detected RTL shunt in 60.1% of patients, while the TTE detected only 37.2%, thus resulting in a statistically significant difference between the two methods (*p* = 0.00012). Also, in this study, there was major accuracy in detecting the large RTL shunt, with a percentage of detection of 42.6% for the raTCD compared to 23% for the TTE. The results of the study underlined the overcoming of the TTE as the gold standard for the diagnosis of RTL shunt, sustaining the possibility of using new advanced models such as raTCD, which showed a sensitivity and specificity of 92% and 87.5%, respectively [142]. PFO may not only cause clinically evident ischemic events but may also be responsible for asymptomatic features. PFO is generally associated with “silent” white matter lesions found in patients with no clinical signs of stroke. Some authors proposed specific patterns of the ischemic lesions, such as the subcortical/periventricular, frontal, or occipital distribution [143,144,145]. A semi-automated AI-derived volumetry software was used for associating the presence of PFO with the neurological features, subdividing patients into those with a history of stroke or not [146]. The study confirmed that PFO can be associated with two different cerebrovascular conditions. The presence of an RTL shunt can identify people at high risk of cerebrovascular events among the general population without any clinically evident neurological signs. Pathophysiologically, silent white matter lesions are linked to reduced cerebrovascular reactivity, which protects against large cerebral infarcts and is associated with clinical conditions such as migraine. The presence of silent white matter lesions is associated with clinical implications; in fact, it is strictly associated with cognitive impairment up to dementia [147,148].

PFO has also been related to transient global amnesia [149], migraine [150], and a higher risk of cerebrovascular events in children with sickle cell disease [151].

Hence, the importance of detecting PFO relates not only to the diagnostic workup of ischemic stroke but also to the evaluation of other neurological syndromes leading to cognitive decline.

### 9.2. Cardiac Valve Embolism

Even though the PFO is maybe the most studied cause of cerebral embolism, other sources have to be sought in the diagnostic workup of ESUS. Cardiac valve disease may be responsible for cerebral vessel occlusion because of the rupture of cardiac vegetations or clotting migrating to the encephalic circle. The risk of cardiac valve-associated embolism is even higher in the case of surgery on the cardiac valve or aorta. In fact, during the procedures of valvuloplasty or replacement, periprocedural ischemic stroke is a frequent complication that can occur. Transcatheter aortic valve replacement is especially burdened by this possibility: the risk of stroke during the procedure ranges from 2% to 6% within 30 days of the procedure [152,153]. For this reason, resources have been spent on inventing devices to reduce the risk of periprocedural events: a filter-based cerebral embolic protection device was developed and is nowadays used to limit cerebral microembolism during transcatheter aortic valve replacement [154]. To quantify the impact of these complications, TCD has been used to monitor cerebral microembolism during these procedures, and the initial stages of applying AI in this field have been taken. Thus, in 2022, Baig et al. [155] applied a novel Robotic Transcranial Doppler (NovaGuide Intelligent Ultrasound, NovaSignal Corp., Los Angeles, CA, USA) with AI to assess the accuracy of this model in recording cerebral scatter and to assess the safety of the procedure of transcatheter aortic valve replacement. Twenty-five patients undergoing transcatheter aortic valve replacement were enrolled and subdivided into two groups using SENTINEL cerebral protection system (Claret Medical, Santa Rosa, CA, USA) a cerebral protection device). A higher number of HITSs were detected in the group without cerebral protection; moreover, the system found that the valve positioning and implantation phase are related to the higher risk of cerebral embolism. Given the difficulties in the easy detection of the risk of embolism during these procedures and the burden of the problem in periprocedural complications leading to disability and mortality, AI-based models will probably increasingly find application in practice, even if more studies need to be carried out.

### 9.3. Atrial Fibrillation

Left auricle ablation is rarely associated with clinically evident thromboembolism, but the incidence of silent periprocedural cerebral microembolism is about 5–15% and about 25% of patients with atrial fibrillation present silent microembolic events not clinically relevant after the first ischemic stroke, and this finding worsens the prognosis of patients, increasing the risk of stroke recurrence [156]. For this reason, some studies have been carried out to evaluate TCD monitoring as a tool for the stratification of patients into classes of risk for the recurrence of cerebrovascular events [157].

TCD has been used to quantify the prevalence of HITSs reaching the cerebral circulation during left atrial ablation procedures. There are two main procedures, the radiofrequency (RFA) “very high-power short duration (vHPSD) ablation” and “pulsed field ablation (PFA)” techniques, which are associated with different risks of embolization.

In a very recent study by Meszaros et al., an ra-TCD was used to associate the type of procedure with the risk of cerebral microembolism, according to the number of HITSs. A total of 26 patients were enrolled: 16 underwent VHPSD, while in 10, PFA was performed. Only gas emboli were detected during the ablation procedure, with a difference that was not significant between the PFA and RFA (246 and 138, respectively, with a *p* value of 0.216). Moreover, a statistically significant difference during PFA was ruled out in the emboli detection between the left and right pulmonary veins, with a higher embolic sign passage in the first compared to the latter (*p* = 0.01). The study proposed a higher incidence of HITSs in the case of PFA compared to RFA, but further investigations need to be carried out to confirm the safety of the RFA on the PFA [158].

Another study has applied an automated robotically assisted ultrasound system to register periprocedural TCD microembolic signs in 22 patients who underwent PFA. An MRI study was also performed to detect the ablation-related acute asymptomatic cerebral embolism. The system revealed that the application of PFA to the right superior pulmonary vein was associated with a higher risk of HITS clusters, which occurred in 38.8% of the population of the study [159]. Thus, the application of AI in this field found more use in evaluating the periprocedural risk of cerebral events, aiming to stratify patients into classes of risk and find a safer procedure to reduce the rate of complications.

### 9.4. Carotid Embolism

Carotid plaques may be one of the sources of embolic stroke if the compositional characteristics make the lesion unstable for rupture. In a study by Markus et al. [160] among 200 recruited patients with significant carotid stenosis, in 89 of them, microembolic signs were detected ipsilaterally. HITSs are associated with a higher recurrence of ischemic events, while antiplatelet therapy reduces the incidence of microembolic signs. Very few studies are available about the application of AI to TCD monitoring for detecting cerebral microembolism from carotid arteries. In a study by Kémeny et al., six patients with carotid disease (one with carotid dissection, two with carotid occlusion with contralateral stenosis, one with asymptomatic high-grade stenosis of the ICA, one with bilateral carotid stenosis, and one with significant stenosis at the origin of the vertebral artery) were subjected to a trained neural network (EMBotec V5.1 One) for the automated detection of HITSs. In this group of patients, 174 signs were detected, but 96 were artifacts, and 24 real HITSs were not identified. The sensitivity of the model was 76.5%, and the positive predictive value was 44.8% [130]. Microembolic fragments can reach the cerebral arteries after procedures aiming to treat significant carotid stenosis. In one study in 2022 [161], eight patients with high-grade carotid stenosis undergoing revascularization were enrolled and submitted to a robotic TCD study. The intraoperative surveillance revealed an average of 117 distal emboli in the ipsilateral MCA within the first 30 min of registration (23.6 ± 0.9 min), with a high prevalence of HITSs during the stent placement and implantation phases. The above was the first study evaluating the application of AI in monitoring microembolism during carotid revascularization. In 2023, Izaguirre et al. [162] applied an automated TCD model called NovaGuide™ 2 Intelligent Ultrasound to detect HITSs in a population composed of patients with symptomatic carotid stenosis of more than 50% or asymptomatic patients with stenosis of at least 60% who underwent endarterectomy or stenting during hospitalization. Among the 20 patients enrolled from July to 12 October 2022, none showed periprocedural HITSs, thus opposing the rate found in other studies, probably due to antiplatelet or anticoagulation therapy. In another more recent study, 92 patients enrolled in two centers (Pavia and Liz) underwent prolonged (>30 min) monitoring using novel ra-TCD (NovaGuide Intelligent Ultrasound) during carotid endarterectomy and mechanical thrombectomy; expert operators performed a parallel manual evaluation to compare the results. The study confirmed the feasibility and applicability of AI-based models to detect microembolic signals and variations in the cerebral blood flow during the intervention on the carotid plaques [163]. In another study [164], intraoperative TCD monitoring using the NovaGuideTM 2 TCD Ultrasound machine (Neura Signal, Los Angeles, CA, USA) was applied to confirm the safety of transcarotid artery revascularization on the CAS and CEA, detecting lower numbers of HITSs during and after the procedure. The detection of the lower number of HITSs in transcarotid artery revascularization lays the groundwork for more studies defining the application and safety of the procedure. The main characteristics of studies applying AI to TCD in RTL shunt are reported in Table 3.

### 9.5. Other Causes of ESUS

Cancer is another important cause of ESUS, occurring in 10% of neoplastic patients [165]. There are several mechanisms based on these events. On the one hand, a mechanical cerebral embolism can occur in tumors located in the cardiac chambers or valves, or the case of tumors of the lung parenchyma involving the pulmonary vessels and reaching, after invading the heart, the cerebral circle [166]. However, other tumors can be associated with embolic stroke because of the overtake of pathways leading to thrombo-inflammation [167,168]. Moreover, the mediastinal neoplasms treated with radiotherapy may lead to cerebral ischemic strokes due to damage to the aortic arch atheromas [169]. Given the increasing incidence of neoplastic diseases, growing interest is placed on applying the TCD study to detect silent microembolic signals in patients with tumors. Several studies have been carried out on this topic [170,171,172], applying transcranial monitoring to detect HITSs and to provide a risk stratification of patients with a higher risk of cerebral impairment and poor prognosis. Severe aortic atheroma represents another cause of embolic ischemic stroke. This hypothesis became outdated after conducting studies that evaluated the presence of HITSs during TCD monitoring in patients with no embolic sources other than aortic atherosclerosis [173]. High-dimensional atheroma in the aortic arch, defined as a dimension > 4 mm, or floating atheromas, determined according to the French classification [174], are associated with a higher risk of ischemic events, representing, thus, another etiology of ESUS. The risk is much higher in the case of surgery on the cardiac or aortic regions, as demonstrated by several studies applying TCD monitoring during the procedures to determine the risk of cerebral microembolism by the detection of the scattering signals [175,176]. No studies about the use of AI-based TCD monitoring are available in this field.

## 10. Monitoring in Acute Neurovascular Care and Non-Invasive Intracranial Pressure Measurement

Neurocritical care units deal with all life-threatening conditions, requiring the continuous monitoring of ventilation and intracranial pressure, the evaluation of hemodynamic parameters, and the monitoring of body temperature. This strict observation derives from the risk of quick clinical worsening in a short time. Most of the conditions treated in Neuro-ICU can lead to intracranial hypertension (ICH), whose management is necessary to prevent cerebral deterioration [177,178]. ICH is generally defined by intracranial pressure (ICP) values above 20 mmHg, which is the conventional threshold of abnormal values. ICP consists of four components responsible for the pattern of ICH. These components are the volume of arterial blood, venous blood outflow, cerebrospinal fluid circulation, and brain volume. Given that the brain volume is fixed, the main variable components responsible for the change in ICP are the cerebral blood flow and the cerebrospinal fluid amount: when one of these increases without a decrease in the other, ICP increases too. This relationship is well-defined considering the formula for the estimation of the CBF; indeed, CBF itself is determined by the input pressure in the form of the mean arterial blood pressure (MAP), the ICP, and the cerebrovascular resistance (CVR) according to the relationship CBF = (MAP—ICP)/CVR [179]. One of the leading causes of ICH is tissue edema occurring after traumatic brain injury [180], but several causes, such as intracranial hemorrhage, hydrocephalus, cerebral neoplasms, and severe ischemic accidents, may increase the ICP, leading to potentially life-threatening complications. Cerebral edema leads to cerebral ischemia and secondary damage, which can be worsened by other variables such as hyperventilation, fever, hyperglycemia, fluid overload, or the high positive end-expiratory pressure levels of the ventilator [181]. Due to the high risk of worsening, ICP monitoring is useful for selecting patients who need further diagnostic studies and immediate therapeutic strategies.

Monitoring ICP usually involves invasive methods, such as epidural, subdural, intraparenchymal, and intraventricular monitors [182]. In recent years, more attention has been given to non-invasive methods to overcome the risk of complications; furthermore, some conditions, such as cerebral malaria, mild trauma brain injury, cerebral tumors, and status epilepticus, are absolute contraindications to invasive monitoring [183], requiring the use of stackable techniques. The use of non-invasive methods finds its application in the case of the contraindications to catheter placement or in the case of idiopathic intracranial hypertension, which rarely requires invasive measurement. In this field, a non-invasive TCD study may be useful for monitoring the ICP, even if it is less accurate than other techniques. Increased ICP can affect the cerebral waveforms and hemodynamic variables detected by TCD, which can aid in the diagnosis of the ICH, thanks to the changes in the vascular circulation which consequently occur for the maintenance of the balance, thanks to the cerebral autoregulation mechanisms. Among the TCD-dependent variables, such as cerebral perfusion pressure (CPP), MFV, EDV, PI, and RI [184,185], PI and CPP have shown the strongest association with changes in ICP after brain injury [184]. PI is calculated from the difference in the systolic and diastolic pressure divided by the MFV; normal values of PI are 0.7 ± 0.3 [186]. On the other hand, CPP derives from the difference between mean arterial pressure and intracranial pressure (ICP), producing a number that may reflect the cerebral perfusion state. Several studies have assessed the applicability of the TCD as a valid tool for non-invasive ICP monitoring. In their study, Bellner et al. measured MFV on the MCA in 81 patients with intracranial disorders; in all patients, the intraventricular catheter was placed to measure intracranial pressure [184]. A significant positive correlation was found between PI and ICP values; the sensitivity and specificity of the measurements were 0.88 and 0.69, respectively, in the case of ICP values ranging from 0 to 20 mmHg, with higher accuracy in the case of ICP> 10 mmHg (sensitivity of 0.91 and specificity of 0.79). The study did not find a significant correlation between the CPP and the PI value, contrary to the data observed in one study by Voulgaris et al. [187]. This study has found a significant inverse correlation between CPP and PI, especially for CPP values < 70 mmHg, helping to identify patients with severe brain injury at higher risk of cerebral ischemia at an early stage. The above data were confirmed in another study by Steiger, in which PI values were increased in the case of brain injury compared to healthy controls; moreover, an association with severe intracranial hypertension or cerebral circulatory arrest has been detected for PI values more than 3 or 6, respectively [188]. Other studies carried out on patients with severe brain injury have supported these data and showed an association between the high PI values with poor outcomes [189,190].

In 2001, Schmidt et al. compared the values of CPP measured in invasive and non-invasive ways to evaluate the applicability of the TCD in estimating cerebral perfusion [191]. The study, conducted on twenty-five sedated patients with brain injury, showed a maximum difference of 13 mmHg between the real and control CPP in 92% of the evaluated patients, with a not-very-wide 95% confidence interval (ranging from 70 to 90 mmHg), confirming the feasibility of TCD application in non-invasive monitoring in patients with ICH. More recently, Zweifel et al. have found a strong correlation between the PI and the ICP/CPP, but with a high predictive value only in the case of ICP > 35 mmHg and CPP < 55 mmHg, while the accuracy decreases for borderline values [192]. However, the wide 95% confidence interval reflects the limitation in the application of the PI-based formula alone as an accurate tool for the diagnosis of ICP. These data were in accordance with the results of the study conducted by Behrens et al. [193]. In this study, PI was calculated in ten patients who underwent a lumbar infusion test, performing concomitant intracranial pressure monitoring. The equation for the determination of the ICP only used the PI (ICP = 23*PI + 14) but showed a 95% confidence interval for a mean value of ICP of 20 mmHg ranging from −3.8 to 43.8 mmHg, suggesting the formula was not able to provide satisfactory results. The reduced accuracy may be due to the effects of several conditions on the determination of PI. First of all, the rigidity of the vascular wall may overestimate the PI’s value in diabetic patients with cerebral microangiopathy [194]. Hemodynamic forces, depending on the cardiac function, may influence the cerebral circulation and the MFV; thus, heart failure can cause the underestimation of the PI [195]. Thus, efforts have been made to improve the diagnostic tools.

### 10.1. TCD-Applied Artificial Intelligence Strategies to Improve Non-Invasive ICP Monitoring

The limitations in the use of TCD for the diagnosis of ICH have led to attempts to develop strategies to increase the accuracy of this tool. In 2012, Sunghan et al. adopted a semisupervised learning algorithm using morphological clustering and the analysis of intracranial pressure pulses (MOCAIP) which was able to extract CBF waveform images from the TCD study [196]. The MOCAIP algorithm [197], consisting of 1435 pulses, constructs one final and valid dominant pulse from the ICP pulse cluster, recognizing the valid and noise-free signals. The dominant pulse contains three peaks, corresponding to the mean ICP. Moreover, a larger distance between the second and third peaks is found in the case of high values of ICP. The proposed method was compared to the PI-based methods: the results have shown that the formulas based only on the PI have less accuracy in reflecting ICH compared to the pulse morphological metrics. The semisupervised algorithm has shown a higher predictive accuracy than the supervised and PI-based methods (92% vs. 82% vs. 59%, respectively). The decision curve analysis has also confirmed the clinical relevance of the method in stratifying the necessity of treatment. Compared to the MOCAIP algorithm, a deep neural network approach based on the direct extraction of TCD-CBF waveforms has been built by Wei et al. for the automated identification of ICH [198]. Datasets collected from 89 patients consisted of 638 images; among these, 90 episodes corresponded to intracranial hypertension (>20 mmHg). This method has been shown to enhance the possibility of ICH monitoring, given the smaller 95% confidence interval in comparison with the MOCAIP (10.6% vs. 20.5%). The difference between the two approaches concerns a preprocessing phase in the case of MOCAIP, which loses hidden patterns that are not visible to the human eye but can be detected by this CNN-based method. These results highlighted the major precision of the method, thanks to the ability to decrease overfitting. However, the approach reached an AUC of 62%, probably because of the small size of the dataset. In 2023, Megjhani et al. proposed a deep learning framework for the automated detection of ICP [199]. The study was conducted on patients with external ventricular drainage admitted to the neurocritical care unit at the Columbia University Irving Medical Center between 2017 and 2022. Patients underwent TCD monitoring to register their cerebral waveforms and flow velocity, electrocardiogram registration, and arterial blood pressure measurement; all the data were digitally collected. The AI-based proposed network consisted of three domains: the Domain Classifier, which can collect ICP dynamics; the ICP Estimator; and the Feature Extractor. The study recruited 13 patients and collected 544,590 data points to build the automated algorithm. The model showed high accuracy, with a maximum error of 5 mmHg compared to the invasive ICP measurement. Thus, constructing a domain adversarial model has shown better performance than existing models, even if more studies need to be carried out to confirm the accuracy of the results. Krieg et al. proposed a classification model that is effective in stratifying patients needing immediate intervention [200]. The study was carried out on twenty-five patients with severe traumatic brain injury under invasive ICP monitoring in the ICU. All the patients underwent transcranial transmission ultrasound, whose images were extracted and validated by a leave-one-subject-out method. The automated model was able to detect ICP over 15 mmHg, with a high sensitivity and predictive negative value (100%), though there was a scarce specificity and predictive positive value (47% and 14%, respectively), showing the ability of the method to rule out patients with high ICP needing further investigations and immediate treatment. In a very recent study, Frigieri et al. [201] proposed the application of an ML model to a novel cranial extensometer device, named the brain4care [B4C] System, for the registration of cerebral waveforms, compared to the TCD images. One hundred and twelve patients were recruited, collecting approximately ~150,000 pulses; 5.36% of the images corresponded to ICP values > 20 mmHg. Thirteen patients underwent concurrent B4C and TCD measurements to validate the model’s accuracy. The model has shown better performance, with a 95% confidence interval for ICP prediction of ±4.17 mmHg for eICP, in comparison with the ±10.78 mmHg for eICPTCD, accounting for a simplified and accurate approach to automated non-invasive ICP monitoring. The reported studies have shown that artificial intelligence may help improve the TCD technique, making it a powerful tool with possible future applications in ICU and non-ICU settings. These promising data need to be validated by more research to obtain a valid model able to improve the detection, prognostic classification, and treatment of ICH.

### 10.2. Optic Nerve Sheath Diameters for Non-Invasive ICP Monitoring

Another application of ultrasound Doppler in the non-invasive identification of intracranial hypertension is the evaluation of the optic nerve sheath diameter (ONSD). Thanks to its feasibility, low cost, and bedside applicability, this method entered clinical practice for ICU monitoring. An increase in ICP leads to the distension of the optic nerve sheath, and so an increase in its diameter [202,203]. Positioning the linear probe on the closed eye, the optic nerve is about 3 mm behind the ocular bulb. The normal value of the transversal ONSD is 5 mm, while values > 5.8 are considered abnormal and may indicate intracranial hypertension. Several studies have found the technique to have good accuracy, suggesting that an ONSD value > 5.7 mm is related to ICP more than 20 mmHg, with a sensitivity of 92% (*p* value of 0.00803) [204]. Due to the limitations of expert operators, AI has also found applications in this field. In 2022, Netterland et al. applied an ML algorithm in their study for the automated segmentation of the ONSD images registered by the ultrasound technique in twenty-five patients with traumatic brain injury [205]. Manual and automated measurements were compared, showing a reasonable agreement in distinguishing high and low ICP values (manual: AUC 0.74, 95% CI 0.58–0.88; automatic: AUC 0.83, 95% CI 0.66–0.93), supporting the possibility to introduce new techniques for ICP monitoring, overcoming the limitations of experienced operators. Subsequently, a study has applied explainable artificial intelligence to minimize human errors in a cohort of 126 patients with SAH, of whom 82 patients presented a high value of ICP (>20 mmHg) and a corresponding ONSD of 0.545 ± 0.08 cm. The application of an AI model helped to increase the diagnostic power of less-used non-invasive techniques, even if some drawbacks, such as the quality of the extracted images or the differences between patients in the same population, may reduce the accuracy of the model [206]. In a very recent study published in 2025, Fu et al. applied AI for the measurement of the ONSD in a population of 199 patients with traumatic brain injury who underwent Doppler ultrasound [207]. Images were extracted, selected by the LASSO regression, and used to construct automated models. A nomogram was built based on integrating clinical, ultrasound, and radiomic features. Different AI methods based on logistic regression, SVM, random forest, and K-Nearest Neighbors were used. Among these, logistic regression has shown the highest accuracy, specificity, and NPV in the validation cohort compared to the other approaches.

The main characteristics of studies applying AI to TCD in ICH are reported in Table 4. The advancement of non-invasive methods for diagnosing and monitoring intracranial hypertension is taking hold, representing a valuable and safe tool comparable to the more harmful invasive techniques. Research in this area needs to be carried out to improve accuracy, and the application of AI in this setting can help to improve the precision of the available tools to better stratify patients based on the risk of complications and to increase safety, reducing the periprocedural risks correlated with the use of invasive methods.

## 11. Transcranial Brain Parenchyma Sonography

TCS allows for the visualization of the structures within the brain through temporal, suboccipital, and orbital windows. It is particularly useful in assessing brain parenchyma, detecting pathological changes, and aiding in the differential diagnosis of neurological disorders [10]. Unlike other imaging modalities such as MRI or CT, TCS offers real-time imaging capabilities, portability, and cost-effectiveness. The primary acoustic window used in TCS is the temporal bone for the visualization of deep brain structures such as the basal ganglia, midbrain, thalamus, and ventricular system. Other acoustic windows include the suboccipital and orbital windows, which allow for the visualization of the posterior fossa and optic nerve, respectively [208]. The technique depends on the differences in echogenicity among various brain structures and pathological formations to detect abnormalities [209]. TCS is widely used by neurologists in the diagnostic workup in the case of movement disorders, cerebrovascular diseases, and space-occupying lesions. In recent years, many studies have tried to apply AI in the ultrasound study of cerebral parenchyma to enhance the sensitivity and specificity of TCS and to find new b-mode hallmarks facilitating the differential diagnosis between several central nervous system diseases.

### 11.1. TCS in the Evaluation of Neurodegenerative Dementias

#### 11.1.1. Parkinson’s Disease and Parkinsonian Syndromes

TCS has been extensively studied in the context of Parkinson’s disease (PD) and atypical Parkinsonian syndromes. In PD, TCS frequently reveals an increased echogenicity of the substantia nigra (SN), a hallmark finding associated with nigrostriatal degeneration. This hyperechogenicity is thought to be due to increased iron deposition and gliosis in the affected region. Studies have demonstrated that SN hyperechogenicity is present in up to 90% of PD patients and can even be detected in asymptomatic individuals at risk of developing the disease, highlighting its potential role in early diagnosis [210].

In contrast, atypical Parkinsonian syndromes, such as multiple system atrophy (MSA) and progressive supranuclear palsy (PSP), show different echogenic patterns. MSA often presents with normal SN echogenicity but demonstrates abnormalities in the lentiform nucleus and brainstem, while PSP is characterized by midbrain atrophy and the reduced echogenicity of the substantia nigra. A recent study introduced the Parkinson’s Disease Denoising and Segmentation Network (PADS-Net) [211], designed to simultaneously denoise and segment transcranial ultrasound images of the midbrain to enhance the accuracy of PD diagnosis. Built upon generative adversarial networks, PADS-Net incorporates a multi-task deep learning framework that optimizes both denoising and segmentation processes for ultrasound imaging. Ultimately, PADS-Net achieved high diagnostic performance, with an AUC of 0.87 for PD detection. In a retrospective study, Kang et al. [212] trained a U-Net model for midbrain auto-segmentation using transcranial sonography images in 584 patients who were suspected of having PD, obtaining a model with good stability, efficiency, and robustness. Another interesting study proposed a DL- DL-residual network model, augmented with attention mechanisms and multi-scale feature extraction to assist in diagnosis with classification accuracy (92.79%), precision (95.42%), and specificity (93.1%) [213]. Moreover, Ding et al. retrospectively compared a deep CNN model with traditional diagnostic methods to predict PD. The model demonstrated good performance in distinguishing between PD patients and normal control subjects with an AUC value of around 90% in the best-performing models [214]. One of the main issues in the ultrasound study of the midbrain is identifying the correct region of interest. A recent study [215] highlights the critical role of region of interest selection in TCS-based computer-aided diagnosis for PD. By comparing three region of interest sizes—the entire midbrain, half of the midbrain, and the substantia nigra —the findings demonstrate that the entire midbrain region provides the highest diagnostic performance. The results suggest that neighboring midbrain areas may exhibit abnormalities not visible to the naked eye, emphasizing the importance of a larger region of interest for more comprehensive feature representation and improved PD diagnosis.

#### 11.1.2. Alzheimer’s Disease and Vascular Dementia

TCS has shown a potential application in detecting alterations associated with Alzheimer’s disease, although its role is less well-established compared to its application in PD. Some studies indicate that TCS can detect reduced echogenicity in the thalamus and hippocampal regions, potentially reflecting atrophy and neurodegeneration in these regions. Additionally, transcranial ultrasound can detect periventricular hyperechogenicity, which may be associated with amyloid deposition and neurodegeneration [216]. Vascular dementia is primarily associated with cerebrovascular pathology, including small vessel disease and strategic infarcts. As mentioned above [89,90], TCS can be used to assess cerebrovascular reserve by evaluating the reactivity of intracranial arteries to CO2 challenges. Increased resistance in the MCA and reduced cerebrovascular reactivity are common findings in patients with vascular cognitive impairment. TCS also aids in detecting periventricular white matter changes by identifying diffuse hyperechogenicity patterns. TCS may show the dilation of the lateral ventricles, a consequence of brain atrophy secondary to chronic ischemic lesions. Furthermore, TCCD can reveal changes in cerebral blood flow dynamics, such as lower MFV in the middle cerebral artery and reduced cerebrovascular reactivity, both of which signal endothelial dysfunction and a heightened risk of cognitive decline [11]. Currently, no known studies have integrated AI and TCS for the diagnosis of Alzheimer’s disease or vascular dementia.

### 11.2. TCS in the Detection of Space-Occupying Lesions

TCS provides valuable information in the identification of intracranial tumors. Tumors typically exhibit distinct echogenic patterns depending on their histopathological characteristics [217]. High-grade gliomas and metastases often appear as hyperechoic masses with irregular margins and increased vascularization detected via Doppler mode. Benign tumors, such as meningiomas, may also present as well-defined hyperechoic lesions with calcifications leading to enhanced reflectivity [218]. TCS can further aid in the differentiation of tumors based on their effect on surrounding structures, such as ventricular displacement and midline shift, which can be observed through changes in brain symmetry. In some cases, TCS-guided, contrast-enhanced ultrasound can improve lesion delineation and vascular characterization [219]. The limited data available in the literature regarding the use of TCS for the diagnosis and monitoring of space-occupying brain lesions has not yet encouraged researchers to apply AI models to enhance the diagnostic accuracy of this technique. However, DL models, particularly CNNs, hold significant potential for this application by enabling automated image analysis, improving lesion segmentation, and enhancing the differentiation between benign and malignant brain tumors. No study combining TCS and artificial intelligence has been conducted to enhance the recognition of cerebral occupying space lesions.

## 12. Application of AI for the Spectral Feature Extraction in Doppler Ultrasounds

The variations in blood flow following the systolic and diastolic phases are shown as a spectral analysis in the form of a sonogram in the Doppler studies. The spectral analysis in Doppler power is the reflection of the changes in the velocity distribution in the vascular districts, allowing for the diagnosis and monitoring of arterial diseases [220,221]. Some parameters are linked to the blood flow changes and have a clinical impact: among these, the RI and PI are used for the evaluation of the Doppler sonograms. Several methods are used to extract features, such as the fast Fourier transform, the autoregressive method, the moving average, and wavelet transform methods, which allow for the construction of the arterial Doppler waveforms. Artificial intelligence (AI) is increasingly applied to spectral feature extraction in Doppler ultrasound to automate and enhance the accuracy of clinically relevant measurements such as blood flow velocity, spectral envelope delineation, and cardiac cycle event detection. Deep learning models, particularly convolutional neural networks (CNNs) and recurrent neural networks (RNNs), have demonstrated good performance in extracting temporal and spectral features from Doppler spectrograms, enabling tasks such as automatic end-diastole detection in cardiac Doppler without the need for ECG signals, with mean errors as low as 14 ms and true detection rates above 97% [222]. AI-based frameworks also facilitate the robust classification and featurization of blood flow signals, including speckle pattern analysis for velocity field estimation, by leveraging machine learning algorithms for dimensionality reduction and feature extraction from ultrasound B-mode images [223]. In arterial disease diagnosis, neural networks trained on feature vectors derived from Doppler signals, using methods such as discrete wavelet transform or eigenvector-based power spectral density estimates, improve classification accuracy and convergence rates [224,225]. Additionally, deep learning models can address technical challenges such as Doppler aliasing in vector flow imaging by segmenting and correcting aliased regions, thus improving the real-time visualization and quantification of complex flow patterns [226]. Artificial neural network architectures have been applied in an attempt to improve the performance of the extraction methods [227], even if few studies are available on the state-of-the-art. ANNs can recognize non-linear models to achieve new patterns for the generalization of the results. Among these, the multilayer perceptron neural network (MLPNN), a non-parametric model analyzing several feature vectors, consists of an input layer, an output layer for the dependent variables, and at least one hidden layer, which contains neurons summing the multiplied input signals producing the output data. The ANNs include the training algorithms to achieve better accuracy. The ophthalmic artery sonogram was used for evaluating the applicability of the MLPNN for the automated extraction of Doppler signals. A total of 214 subjects have been recruited and underwent ophthalmic arterial Doppler ultrasonography, subdivided into subgroups (stenosis, Behcet disease, uveitis, healthy controls), resulting in a 129-point logarithm of the power spectral density values obtained by several feature extractions. A total of 256 samples were obtained by each ophthalmic arterial Doppler signal frame, and seven levels of the 254 detail wavelet coefficients were computed. Four MLPNNs were constructed and trained with five different algorithms to cross-validate the method, which showed an accuracy of more than 85% for the classification of the ophthalmic arterial Doppler signals. MLPNN was also applied for the classification of the carotid artery signals, showing the same accuracy [224]. A signal-to-noise ratio saliency measure was used to analyze the inputs in the MLPNNs, showing the higher accuracy of the classification using salient input features, increasing the diagnostic power of these automated methods [228].

More recently, ANNs were applied to recognize the different spectrograms in the common carotid, common femoral, and popliteal arteries on the basis of the maximum frequency envelopes, and were used to create sets of training and testing vectors. The method showed a high accuracy, accounting for a helpful tool to differentiate similar flow profiles and to recognize the onset of arterial diseases early [227].

In conclusion, ANNs can guarantee an accurate feature extraction by the usage of the discrete wavelet coefficients, drawing a better representation of the Doppler signals and allowing for an easier interpretation of the arterial sonograms for the diagnosis of vascular diseases.

## 13. Discussion

**Figure 2:**
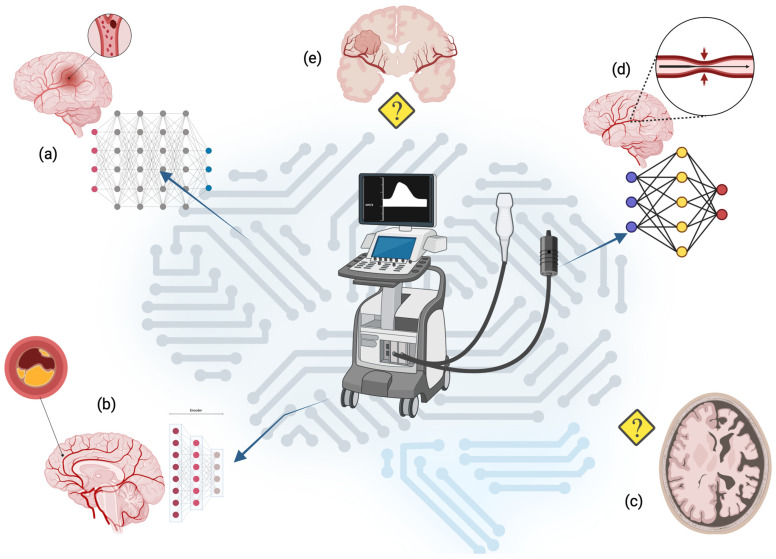
Current application of artificial intelligence in neurosonology. Machine learning, deep learning, and convolutional neural network algorithms have been successfully applied to Transcranial Doppler and Transcranial Color-Coded Doppler for the diagnosis of cerebral microembolization (**a**), intracranial stenosis (**b**), and vasospasm secondary to subarachnoid hemorrhage (**d**). In contrast, the application of artificial intelligence to transcranial sonography for the evaluation of parenchymal diseases, such as dementia (**c**) and space-occupying lesions (**e**), has not yet been explored, but it represents a promising area for future research and clinical development.

The application of AI to neurosonology, particularly TCD and TCS, represents a potentially transformative advancement for enhancing the effectiveness, reliability, and accessibility of these techniques (Table 5). Machine learning, deep learning, and convolutional neural network algorithms have been effectively utilized in the analysis of Transcranial Doppler and Transcranial Color-Coded Doppler data for several conditions. Conversely, the application of artificial intelligence techniques to transcranial sonography for the assessment of parenchymal brain disorders, such as dementia and space-occupying lesions, remains largely unexplored. Nonetheless, this area holds significant potential for future research and clinical innovations (Figure 2).

Although TCD and TCS are well-established tools for assessing cerebral hemodynamics, detecting microembolic signals, and studying brain parenchyma, they are still limited by several practical challenges. These include difficulties in locating optimal acoustic windows through the skull, the precise identification of intracranial arteries, and the steep learning curve required for proper technique—factors that make these modalities highly operator-dependent. The integration of AI, especially models based on CNNs, may provide a concrete solution to these limitations. One of the most promising applications lies in the automatic detection of microembolic signals. At present, identifying these signals with TCD requires considerable clinical expertise, as they can vary in intensity and are often mistaken for artifacts or background noise. A properly trained AI algorithm, using extensive and validated datasets, can significantly enhance the sensitivity and specificity of detection. This would minimize subjectivity in interpretation and ensure greater standardization across operators and institutions. Moreover, such an automated system could allow for continuous, real-time monitoring, even in emergency settings or stroke units, thereby supporting faster and more accurate clinical decision-making. Another area where AI may bring tangible benefits is the automatic identification of intracranial arteries during TCD examination. Proper insonation of vessels such as the middle cerebral artery, anterior cerebral artery, and posterior cerebral artery can be technically challenging, particularly for less-experienced operators. Intelligent algorithms embedded within ultrasound systems could guide the user with real-time feedback on probe positioning and signal quality, streamlining the identification of target vessels. Similarly, the localization of optimal transtemporal windows, often complicated by anatomical variability or the thickness of the temporal bone, could be assisted by predictive systems capable of analyzing ultrasound images and recommending the best insonation point. Looking forward, AI integration into transcranial parenchymal imaging could open new diagnostic horizons. CNNs, already widely employed in medical image analysis within neuroradiology, could be adapted to automatically classify space-occupying lesions such as tumors or hematomas and to identify early patterns characteristic of neurodegenerative diseases like Parkinson’s disease or dementia. CNNs are particularly well-suited to this task due to their ability to learn and recognize complex visual features, even in the presence of morphological variability or image artifacts. As such, AI-enhanced sonographic evaluation of the brain parenchyma could improve diagnostic accuracy, assist in longitudinal monitoring of lesions, and enable earlier diagnosis in at-risk populations. In the future, the integration of artificial intelligence into neurosonology could not only offer a means of overcoming current technical and operational limitations but also mark a decisive step toward more precise, standardized, and widely accessible cerebral diagnostics. The development of intelligent ultrasound devices with autonomous analytical capabilities may facilitate the broader use of TCD and TCS in clinical practice, optimize diagnostic workflows, and ultimately enhance care and recognize new hallmarks for cerebrovascular and neurological disorders, especially neurodegenerative disorders and dementias.

### AI Use-Case in a Clinical Scenario and Its Limitations

The current literature demonstrates a range of AI applications in neurosonology, representing a valuable tool for several cerebral diseases. In the screening setting, AI algorithms help to indirectly diagnose intracranial stenosis by the changes in cerebral blood flow secondary to intracranial stenosis, which cannot be directly visualized, to prevent the progression of vascular damage to occlusion. ML algorithms have been applied in the monitoring of patients with subarachnoid hemorrhage, where the assessment of MFV variations allows for the early identification of patients who will develop vasospasm, helping to identify patients needing early treatment to avoid chronic brain damage. After trauma, patient monitoring allows for the identification of changes in cerebral perfusion pressure, hemodynamic parameters, or increased ONSD values as early indicators of intracranial hypertension. In cerebral microembolism, ML-tools for the detection of HITSs may help to assess the risk of microembolization responsible for silent stroke in patients with PFO, and also the risk of cerebral embolization during ablation interventions in patients with atrial fibrillation or during endarterectomy, allowing for the identification of procedures related to an increased periprocedural risk of acute cerebrovascular events and therefore an outline of the safest strategies. The application of artificial intelligence to the evaluation of cerebral parenchyma can also be a useful tool in the diagnostic workup of neurodegenerative pathologies, in which the change in brain structures due to neuronal depletion is associated with a change in echogenicity detected at TCD. The data derived from both retrospective studies and prospective cohort studies were used to demonstrate and validate the accuracy of the results obtained by applying artificial intelligence in various clinical scenarios. To date, the results are promising for the future application of these methods in clinical practice. Some of these systems have already been approved: in 2020, the FDA approved the LucidTM M1 Transcranial Doppler Ultrasound System^®^ and NeuralBotTM System as robotically assisted ultrasound systems for the measurement of the cerebral blood flow velocity. However, there are some additional limitations to adopting these new instruments in clinical practice. Firstly, the costs of applying AI-based tools could increase health expenditure, given the need for reimbursement by the health system to ensure their adequate use on a large scale. In addition, not all systems have yet been regularly approved. Finally, specialized staff need to be trained to use these new tools. Overcoming these barriers is necessary for applying these new tools in clinical practice, aiming to improve TCD performance in the diagnosis and monitoring of various clinical scenarios.

## 14. Limitations

Despite the comprehensive and methodologically rigorous approach adopted in this scoping review, several limitations must be acknowledged. First, the exclusion of non-English language publications may have led to the omission of relevant studies, particularly those conducted in regions with active research in neurosonology but limited English-language dissemination. Second, the heterogeneity of the included studies in terms of clinical focus, AI algorithms used, sample sizes, and validation strategies posed challenges for direct comparison and precluded meta-analytic synthesis. Furthermore, the lack of standardized reporting practices across studies, including the inconsistent disclosure of performance metrics and external validation procedures, limits the ability to draw definitive conclusions regarding the generalizability and clinical utility of the proposed models. The predominance of datasets from North America and East Asia also raises concerns about population representativeness and the global applicability of AI-driven neurosonology tools. Additionally, no protocol was pre-registered for this review, potentially introducing selection and reporting bias.

## 15. Conclusions

In conclusion, TCD, TCCD, and TCS represent valuable, non-invasive, and radiation-free tools for the assessment of cerebral hemodynamics, microembolic signal detection, and the evaluation of brain parenchyma. However, these modalities require a higher level of operator expertise and present a steeper learning curve compared to other ultrasound techniques. Additionally, suboptimal acoustic windows due to cranial bone structures can further limit their application. The integration of artificial intelligence offers a promising solution to these challenges. In particular, AI can assist clinicians in optimizing arterial insonation during TCD and in enhancing the accuracy and consistency of microembolic signal detection. The implementation of AI-driven algorithms in future TCD systems is highly desirable and may significantly improve diagnostic performance, reproducibility, and accessibility in neurosonology. The development of robust, generalizable AI models and the integration of multimodal imaging data promise to enhance diagnostic and prognostic capabilities in TCD and ultrasound. By bridging the gap between technological innovation and clinical utility, AI has the potential to reshape the landscape of neurovascular and diagnostic imaging, driving advancements in personalized medicine and improving patient outcomes.

## Figures and Tables

**Table 1:** Employment of AI-applied TCD: Characteristics of clinical studies regarding intracranial atherosclerotic stenosis (ICAS).

	Reference	Year of Publication	Sample Size (N° of Patients)	Data-Source Origin	Algorithm	Validation Strategy	Performance Metrics
ICAS	[69] Hsu, K-C et al.	2020	8211	Single-center cohort	SVM	Leave-one-out (LOO) cross-validation with ten bootstrap samplings	Sensitivity: 71.7–100% Specificity: 88.9–100%
	[71] Mei, Y. J. et al.	2022	276 (203 healthy individuals and 73 patients with ICAS)	Single-center cohort	CNN	Comparison with healthy group	Sensitivity: 80% Specificity: 83%
	[72] Yeh, C.-Y. et al.	2022	538	Public registry (DICOM)	ML model	10-fold cross-validation	Accuracy: 67–86%
	[73] Xu, D. et al.	2024	1729	Single-center cohort	DL (model VGG16)	Combined dataset consisting of TCD examination images from hospitalized patients (dataset1) and a population undergoing routine medical check-ups (dataset2)	Accuracy: 85.67 ± 0.43 Sensitivity: 83.60 ± 1.60 Specificity: 87.73 ± 1.47
	[74] Nisha, N.N. et al.	2023	18 (6 healthy individuals and 12 patients)	Public registry	Self-Organized Operational Neural Network (Self-ONN)-based deep learning model: Self-ResAttentioNet18	5-fold cross-validation	Accuracy: 96.05% Specificity: 96% ROC curve: 0.99

**Table 2:** Employment of AI-applied TCD: Characteristics of clinical studies regarding subarachnoid hemorrhage (SAH).

	Reference	Year of Publication	Sample Size (N° of Patients)	Data-Source Origin	Algorithm	Validation Strategy	Performance Metrics
SAH	[117] Elzaafarany, K. et al.	2018	160	Public registry	Pattern-recognition ML model	Error analysis was performed by using precision and recall measures	Sensitivity: 87.5% Specificity: 89.7%
	[118] Kumar, G. et al.	2017	267	Public registry	ML model	Cross-validation technique used for training a classifier using 50% of the data	Sensitivity: 78% Specificity: 91%
	[120] Clare, K. Et al	2022	12	Single-center cohort	NovaGuide Model	Comparison with CTA studies	Sensitivity: 83% Negative predictive value: 90% Positive likelihood ratio: 8.75
	[123] Kim, Y-G. et al.	2023	19	Single-center cohort	DL-based convolutional layer-based neural network	Training dataset comprised 1727 Doppler wave files; the remaining 565 files were evaluated for validation using the proposed classifier	Accuracy: 90% Sensitivity:90%
	[125] Mohammadzadeh, I. et al.	2025		Public registry (metanalysis of eight studies)	ML algorithm		Sensitivity: 79% Specificity: 78% AUC: 0.85

**Table 3:** Employment of AI-applied TCD: Characteristics of clinical studies regarding right-to-left shunt (RTLS).

	Reference	Year of Publication	Sample Size (N° of Patients)	Data-Source Origin	Algorithm	Validation Strategy	Performance Metrics
RTL SHUNT (patent foramen ovale)	[137] Rubin, M. N. et al.	2023	129	Multi-center cohort	Robot-assisted TCD (raTCD)	Comparison with TTE	Sensitivity: 64%
	[139] Chang, I. et al.	2024	212	Single-center retrospective cohort study	raTCD	Comparison with TEE	Sensitivity: 91–100% Specificity: 78–100%
	[141] Shah, R. et al.	2025	148	Single-center cohort	raTCD	Comparison with TTE bubble studies, performed by certified ultrasonographers and read by blinded level III echocardiography board-certified cardiologists	Sensitivity: 95% Specificity: 88.9%
RTL SHUNT (cardiac valve embolism)	[154] Baig, A. et al.	2022	8	Single-center cohort	TCD robot head-brace system	Linear regression model	-
RTL SHUNT (atrial fibrillation)	[157] Meszaros, H. et al.	2024	26	Single-center cohort	raTCD	Post-operative cranial MRI exams were performed; the MES load from the different pulmonary veins was compared using the Wilcoxon test and Bonferroni correction	Statistical difference comparing pulmonary veins, with *p* value < 0.01
	[158] Della Rocca, D. G. et al.	2023	20	Single-center cohort	raTCD	MRI sequences 24–48 h post-ablation	Statistical difference comparing procedures, with *p* value < 0.01
RTL SHUNT (carotid embolism)	[162] Fattorello Salimbeni, A. et al.	2024	92	Single-center cohort	NovaGuide™2 Intelligent Ultrasound	Comparison with parallel manual evaluation	High accuracy

**Table 4:** Employment of AI-applied TCD: Characteristics of clinical studies regarding intracranial hypertension (ICH).

	Reference	Year of Publication	Sample Size (N° of Patients)	Data-Source Origin	Algorithm	Validation Strategy	Performance Metrics
ICH	[197] Wei, M. et al.	2025	89	Single-center cohort	MOCAIP algorithm	10-fold cross-validation	ROC curve (AUC) of 96%
	[198] Megjhani, M. et al.	2023	13	Single-center cohort	ML model	Leave-one-session-out cross-validation technique	High accuracy
	[199] Krieg, S. M. et al.	2024	25	Single-center cohort	ML model	Comparison with invasive monitoring	Sensitivity: 100 Specificity: 47% NPV: 100% PPV: 14%
	[206] Fu, Z. et al.	2025	199	Retrospective observational single-cohort study	SVM	LASSO regression	AUC: 0.840 Accuracy: 0.853 Sensitivity: 0.69 Specificity: 0.89 PPV: 0.800 NPV: 0.858

**Table 5:** Advantages of the application of AI in TCD in clinical practice.

	Aim of the Use of AI-Applied Methods	Parameters	Results	Benefits	Limitations
ICAS	Evaluation of hemodynamic variables to indirectly identify intracranial stenosis to select the population needing to undergo further examinations	FV, PSV, EDV, MFV	Reduction in FV; increase in PSV, EDV and MFV; reduction in PI	Reducing the ionizing radiation exposure	Accuracy related to the degree of stenosis, with weak accuracy in the case of mild stenosis
SAH	Early detection of cerebral vasospasm by variations in cerebral circulation	MFV, intra-extracranial MFV ratio; PSV and EDV	MFV > 120 cm/s and MFV ratio > 3 is indicative of vasospasm; increase in PSV and EDV	Easy monitoring; high-grade vasospasm is demonstrated by TCD findings 24–48 h before the appearance of clinical symptoms	Inability to insonate intracranial vessels in 10% to 20% of patients, spatial resolution of TCD limited for the posterior circulation
RTL SHUNT (PFO)	Detection of HITSs for non-invasive diagnosis of PFO	Microembolic signals	High-intensity transient signals on the spectrogram	More feasible, superior sensitivity compared to TTE and TEE; more cost-effective than echocardiography; estimation of the PFO is largely on the basis of the HITS numbers; fewer false negatives; quantification of the RLS severity conducted by using the Spencer Logarithmic Grading Scale (SLS)	TCD with bubble injection is more sensitive but less specific than TTE and TEE with bubble studies
RTL SHUNT (cardiac valve embolism, atrial fibrillation, and carotid embolism)	Detection of cerebral HITSs to evaluate the risk of periprocedural stroke	Microembolic signals on the MCA	The number of HITSs is associated with the risk of symptomatic cerebral ischemia	Intraoperative non-invasive monitoring, assessment of the safety of the procedures	Expert operators, good temporal windows, maintaining the head position
ICH	Non-invasive detection and monitoring of ICH	Evaluation of the CBFV waveforms and PI; ONSD measure	Three-peak CBFV waveform and ONSD > 5 mm are abnormal	Feasibility in patients with contraindications to lumbar puncture, repeatable examination for continuous monitoring in ICU settings	Expert operators, good temporal windows, maintaining the head position
NEURO-DEGENERATIVE DEMENTIA	Recognizing specific patterns in the echogenicity of the brain structures to help diagnosis	SN hyper-echogenicity is present in up to 90% of PD patients	Autosegmentation of the cerebral regions with different echogenic patterns is associated with specific diseases	Non-invasive instrument for early diagnosis of neurological decline and cognitive impairment; other acoustic windows include the suboccipital and orbital windows, which allow for the visualization of the posterior fossa and optic nerve, respectively	Need for expert operators

CBFV: cerebral blood flow velocity, EDV: end diastolic velocity, FV: flow velocity, HITSs: high-intensity transitory signals, ICAS: intracranial atherosclerotic stenosis, ICH: intracerebral hemorrhage, ICU: intensive care unit, MCA: mean cerebral artery, MFV: mean flow velocity, ONSD: optic nerve sheath diameter, RTL: right-to-left, PD: Parkinson’s disease, PFO: patent foramen ovale, PI: pulsatility index, PSV: peak systolic velocity, SN: substantia nigra, TCD: Transcranial Doppler, TEE: transesophageal echocardiography, TTE: transthoracic echocardiography.

## Supplementary Materials

The following supporting information can be downloaded at: https://www.mdpi.com/article/10.3390/bioengineering12070681/s1, Preferred Reporting Items for Systematic reviews and Meta-Analyses extension for Scoping Reviews (PRISMA-ScR) Checklist.

## Abbreviations

The following abbreviations are used in this manuscript:

AI: artificial intelligence; ANNs: artificial neural networks; AUC: area under the curve; ACA: anterior cerebral artery; BA: basilar artery; CNNs: convolutional neural networks; CPP: cerebral perfusion pressure; CTA: computed tomography angiography; DL: deep learning; EDV: end-diastolic velocity; ESUS: embolic stroke of undetermined source; FDA: Food and Drug Administration; FFT: fast Fourier transform; FV: flow velocity; GANs: generative adversarial networks; HITSs: high-intensity transient signals; ICAS: intracranial atherosclerotic stenosis; ICH: intracranial hypertension; ICP: intracranial pressure; ICU: intensive care unit; MAP: mean arterial blood pressure; MCA: middle cerebral artery; ML: machine learning; MDV: minimum diastolic velocity; MFV: mean flow velocity; MCFV: mean cerebral flow velocity; MOCAIP: morphological clustering and analysis of intracranial pressure pulses; MRA: magnetic resonance angiography; MSA: multiple system atrophy; NLP: natural language processing; ONSD: optic nerve sheath diameter; PCA: posterior cerebral artery; PFO: patent foramen ovale; PI: pulsatility index; PSV: peak systolic velocity; raTCD: robot-assisted TCD; RFA: radiofrequency; PD: Parkinson’s disease; PFA: pulsed field ablation; PSP: progressive supranuclear palsy; RTL: right-to-left; RI: resistance index; RNNs: recurrent neural networks; SAH: subarachnoid hemorrhage; SN: substantia nigra; TCD: Transcranial Doppler; TCCD: Transcranial Color-Coded Doppler; TCS: transcranial sonography; TEE: transesophageal echocardiogram; TTE: transthoracic echocardiogram; SVM: support vector machine; VA: vertebral artery.
